# From task-specific networks to foundation models in diabetes care: a critical review of opportunities, evaluation gaps, and safety frontiers

**DOI:** 10.3389/frai.2026.1983619

**Published:** 2026-10-28

**Authors:** M. Nanda Kishore, C. Navaneethan

**Affiliations:** School of Computer Science Engineering and Information Systems, Vellore Institute of Technology, Vellore, Tamil Nadu, India

**Keywords:** clinical evaluation, continuous glucose monitoring, diabetic retinopathy, electronic health records, foundation models, large language models, model calibration, self-supervised learning

## Abstract

Artificial intelligence in diabetes care has historically relied on task-specific supervised learning, such as bespoke networks for retinopathy grading, kidney disease prediction, and glucose forecasting. The emergence of foundation models (FMs: large models pretrained on broad data, typically with self-supervision, and adapted to many downstream tasks) is displacing this paradigm across diabetes-relevant modalities: ophthalmic models such as RETFound, pretrained on 1.6 million retinal photographs, have been reported to outperform task-specific networks on retinopathy grading and systemic disease prediction, especially when labeled data are scarce; transformer-based time-series models are replacing long short-term memory (LSTM) pipelines for continuous glucose monitoring (CGM) forecasting; clinical large language models (LLMs) such as Med-PaLM and GPT-4 attain expert-level scores on medical question-answering benchmarks; and generative models supply synthetic CGM and electronic health record (EHR) data. This review provides a critical, modality-organized synthesis of this transition. We (i) formalize a three-era taxonomy: rule-based risk scores (era I), task-specific deep learning (era II, 2016–2020), and foundation models (era III, from 2021), and map the diabetes literature onto it; (ii) survey foundation-model architectures, pretraining objectives, and adaptation strategies across retinal imaging, CGM, longitudinal EHR, and multimodal data; (iii) expose an evaluation crisis: benchmark contamination of LLM medical evaluations, a near-universal absence in the literature we reviewed of external and prospective validation for retinal and CGM foundation models, miscalibration under dataset shift, and, to our knowledge and within the reviewed literature (January 2016–December 2025; negative findings are time-sensitive), the absence of any randomized evaluation of a foundation model in a diabetes workflow; and (iv) analyze safety risks: hallucinated insulin-dosing advice, automation bias, inherited algorithmic inequity, and privacy exposure during adaptation. We conclude that the decisive open problem is no longer representation learning but grounded, calibrated, causally informed deployment, requiring contamination-resistant evaluation, prospective trials, and hybrid architectures coupling foundation-model representations with treatment-effect estimation.

## 1 Introduction

### 1.1 The paradigm shift

Diabetes remains one of the largest chronic disease burdens worldwide (International Diabetes Federation, 2021). For thirty years, clinical risk prediction in diabetes was dominated by era-I artifacts, part of the longer arc of machine learning’s entry into clinical prediction and medicine (Chen and Asch, 2017; Topol, 2019): fixed-weight scoring functions such as the UKPDS risk engine and the DCCT-derived retinopathy progression models, whose coefficients were estimated once, on small cohorts, from hand-engineered features (Stevens et al., 2001). Era II (roughly 2016–2020) replaced these with task-specific deep networks trained end-to-end on labeled data for a single endpoint. Its emblematic success was Gulshan et al.’s convolutional network for diabetic retinopathy (DR) grading (Gulshan et al., 2016), soon followed by comparable CNN-based DR classifiers (Gargeya and Leng, 2017), and then the first autonomous AI diagnostic authorization in any field of medicine, IDx-DR, by the US FDA in 2018 (US Food and Drug Administration, 2018). Era II proved that deep learning could match specialists on narrow, well-benchmarked imaging tasks.

Era III rests on a different premise, so we first define our terms—operational definition. A foundation model (FM) is a model that meets three criteria: (i) it is pretrained on broad data, in practice of order 10^6^ or more units (images, signal windows, patient records, or text tokens; Table 1), using a self-supervised or otherwise task-agnostic objective rather than a single clinical label; (ii) a pretrained backbone is adapted to multiple downstream tasks by linear probing, fine-tuning, in-context learning or retrieval augmentation; and (iii) transfer is demonstrated to at least one task, site, or population absent from pretraining (Bommasani et al., 2021; medical instantiations are surveyed by Moor et al., 2023). The scale criterion is a guide, not a cut-off. Models trained end-to-end for a single endpoint, however large, and supervised ImageNet-initialized networks are not FMs here; they serve as era-II or hybrid baselines. Dating. We date era III from 2021, the year the paradigm was formalized and its enabling methods matured; the justification for this convention rather than a sharp boundary is given in Section 3.1.

**Table 1:** Three-era taxonomy of AI in diabetes care.

Dimension	Era I: risk scores	Era II: task-specific DL	Era III: foundation models
Knowledge source	Hand-estimated coefficients	Task labels	Self-supervised pretraining at scale
Representative artifact	UKPDS risk engine	Gulshan et al. CNN (DR)	RETFound; Med-PaLM 2; GPT-4
Typical data per model	10^2^–10^4^ patients, tabular	10^4^–10^6^ images/series, single modality	10^6^–10^9^ units, multi-task corpora
Label cost per new task	Low (refit)	High (expert annotation)	Low–zero (probe/finetune/in-context)
Task generality	One endpoint	One endpoint	Many endpoints per pretrained backbone
Calibration behavior	Explicit (risk tables)	Often unexamined	Often miscalibrated; prompt-sensitive
Auditability	Full	Partial (post-hoc XAI)	Structurally limited (opaque corpora/scale)
Failure mode	Feature blindness	Distribution shift	Shift + hallucination + contamination
Regulatory precedent	Established (SaMD Class II routes)	IDx-DR (*De Novo*, 2018)	None identified for autonomous FMs in diabetes

The premise matters clinically because the binding constraint of era-II diabetes AI was precisely the labels: expert-graded fundus images, adjudicated CKD endpoints, and physician-labeled ulcer photographs. If general visual, temporal, and linguistic representations can be learned without task labels and transferred to diabetes tasks, the economics (and failure modes) of clinical AI change fundamentally.

Three developments define the era-III frontier for diabetes:

- Ophthalmic foundation models: RETFound (Zhou et al., 2023), a Vision Transformer (ViT-Large) pretrained via masked image modeling (MAE-style) on 1.6 million retinal photographs, was reported by its developers to outperform task-specific CNNs and transfer-learning baselines on DR grading, retinal disease prognosis, and (remarkably) prediction of systemic disease (incident myocardial infarction, heart failure, stroke) from retinal images alone. The retina is a uniquely diabetes-relevant organ: an FM of the retina is, in effect, an FM of the diabetic microvascular state. Independent comparisons are more mixed: the advantage is clearest when labeled data are scarce (Section 4.1.3).
- Clinical large language models: Med-PaLM reached 67.6%, and Med-PaLM 2 reached 86.5% on MedQA, whereas GPT-4-family models surpassed passing thresholds across medical licensing-style benchmarks covering diabetes pharmacotherapy, complication screening, and guideline-content questions (Singhal et al., 2023a, b; Nori et al., 2023). Diabetes management content is central to these benchmarks, and LLMs are already being deployed as patient-facing diabetes chatbots, a deployment class with, to our knowledge, essentially no randomized safety evidence.
- Time-series and multimodal foundation models: Pretrained transformers for physiological time series are being applied to CGM streams for forecasting, hypoglycemia prediction, and glycemic variability representation (e.g., Nie et al., 2023; Zerveas et al., 2021). Multimodal models align retinal, EHR, and textual modalities to produce unified patient representations. While existing reviews in this space (e.g., Deng et al., 2025) organize AI-in-diabetes-care around the patient journey (identification, prediction, assistance, education, and support), this review is organized around the foundation-model paradigm shift: what has changed architecturally and epistemically as the field moves from task-specific supervision to large-scale pretraining, and what evidentiary standard has that shift and has not met?

### 1.2 Why diabetes is the critical test case for era-III clinical AI

Diabetes is arguably the most demanding (and most informative) test case for FMs in medicine, for four structural reasons:

- Modality breadth: Few other chronic diseases generate comparable longitudinal multimodal data per patient: serial retinal imaging, CGM with 288 readings/day, months of continuous insulin dosing (in T1DM), dense laboratory panels, wearables, and structured EHR narratives. A paradigm that claims modality generality must prove itself across this breadth (Figure 1).
- Label economics: The highest-value diabetes prediction targets (incident proliferative retinopathy, eGFR slope decline, foot-ulceration onset) are expensive, delayed, and imbalanced: exactly the regime in which label-efficient pretraining should show the largest advantage and exactly where era-II supervised learning plateaued.
- Regulatory precedent: Diabetes hosts the first FDA-authorized autonomous AI diagnostic (IDx-DR, 2018), and other autonomous DR screening systems (for example, EyeArt and AEYE-DS) have since been cleared; to our knowledge, all are task-specific era-II systems. The regulatory pathway for era-III models (which are non-deterministic, adaptable, and sometimes closed-weight) is contested terrain, and diabetes applications will be either early casualties or early precedents. Existing clinical decision support software guidance (US Food and Drug Administration, 2022) was not written with this model class in mind.
- Direct-patient-facing risk: Unlike a radiology triage tool, LLM-based diabetes assistants interact directly with patients, making decisions about insulin, diet, and hypoglycemia. The gap between benchmark performance and safe autonomous operation is widest (and least forgiven) here.

**Figure 1:**
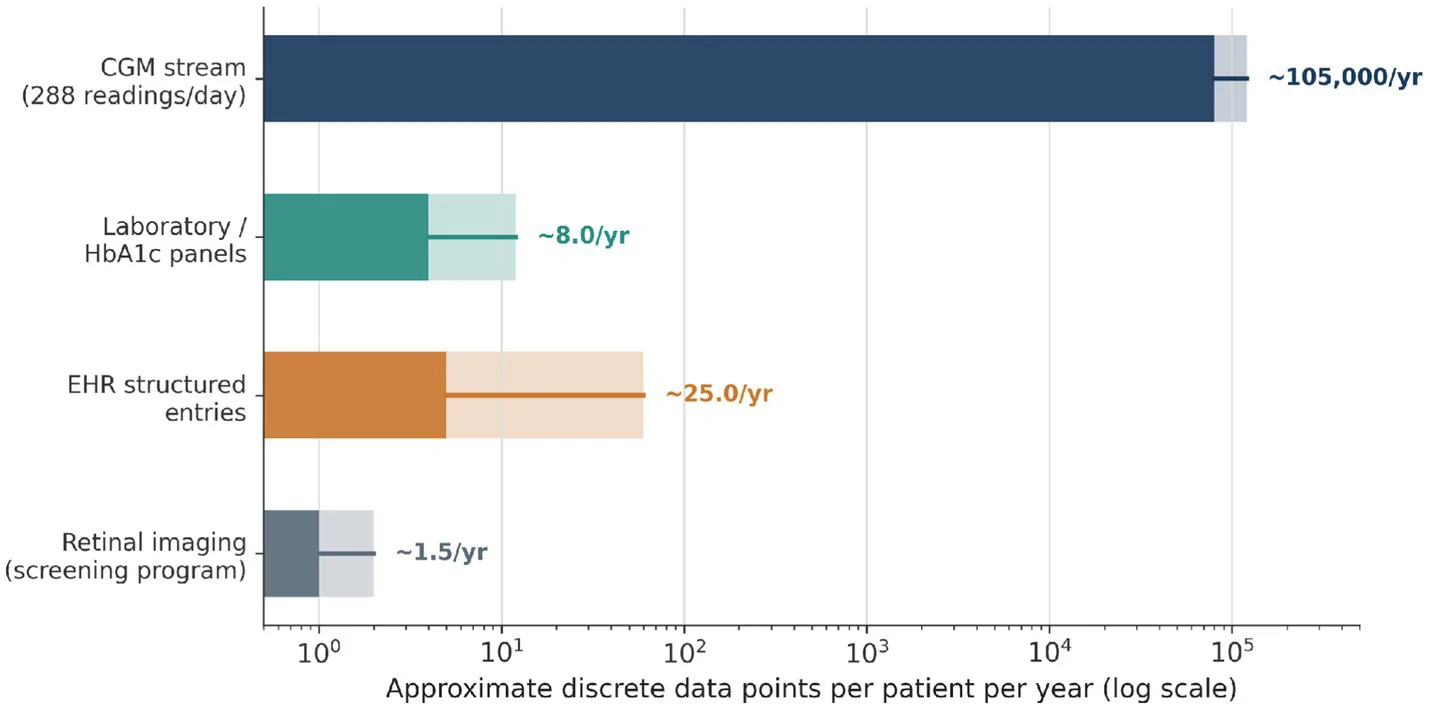
Illustrative data density by diabetes-relevant modality. Approximate number of discrete data points collected per patient per year for retinal imaging, structured EHR entries, laboratory/HbA1c panels, and CGM streams (288 readings/day), on a log scale; the chart illustrates the modality-breadth argument for FMs in diabetes care. Source: Authors’ order-of-magnitude estimates synthesized from the figures cited in Section 1.2; conceptual illustration, not a harmonized empirical dataset.

### 1.3 The argument of this review

The thesis of this review is that FMs invert the central bottleneck in diabetes AI while introducing three new unsolved problems:

- Representation is no longer the bottleneck; evaluation is: Era-II evaluation culture (a public benchmark, an AUROC, and a holdout split) is inadequate for models whose pretraining corpora are opaque, whose benchmarks are plausibly contaminated, and whose stochastic outputs vary with prompting. Section 6 shows that this failure is already pervasive.
- Capability is no longer the bottleneck; grounding is: A model that attains 86.5% on MedQA has not demonstrated that it can correctly advise a specific patient on a specific sulfonylurea taper. The missing layer is causal, patient-specific grounding, and Section 7 shows that its absence carries concrete safety consequences.
- Deployment is no longer hardware-limited but governance-limited: Adaptation, monitoring, and liability frameworks for models that drift, are updated silently by vendors, or generate confident hallucinations remain immature; Section 7 maps them.

To our knowledge, we are among the first reviews to synthesize the foundation-model transition for diabetes care across all modalities and to organize its critique around an evaluation–capability–grounding triad. Our intended readership is dual: computer scientists entering the clinical FM space (who need the diabetes-specific instantiation of architectures, datasets, and pitfalls) and clinical informaticians (who need a technically rigorous account of what changed under the hood).

## 2 Review methodology

### 2.1 Scope and review design

This is a critical narrative review, following SANRA guidance for narrative reviews (Baethge et al., 2019). We deliberately adopt a “critical review” consistent with Grant and Booth’s typology (Grant and Booth, 2009): our contribution is not an exhaustive enumeration but a conceptual synthesis of the three-era taxonomy, onto which the literature is mapped and critiqued. It is not a systematic review (see non-systematic design below).

Literature window: January 2016 (era-II inflection: Gulshan et al., 2016) through December 2025, with an explicit cutoff declared here; the FM literature moves monthly, and we discuss post-cutoff trajectory qualitatively only.

#### 2.1.1 Scope of negative claims

All negative findings in this review (for example, the absence of L1 evidence, of randomized evaluations, published calibration curves, or externally validated multimodal diabetes FMs) describe the literature we identified within the window above (to December 2025), not the field as a whole. Because the search was purposive and drew on a limited set of sources (Section 2.2), an absence does not prove that no such evidence exists; each should be read as “to our knowledge, as of December 2025” and is time-sensitive. Landmark studies known to the authors (for example, RETFound, Med-PaLM, and the GPT-4 technical report) are discussed and graded in Sections 3–5 for context.

#### 2.1.2 Non-systematic design

Because the literature was gathered using narrative-review judgment rather than a systematic protocol, no review protocol was registered. The review does not report the numbers of records identified, screened, excluded, or included; a study-selection flow diagram; a complete list of included studies; or reviewer-agreement statistics. No formal quality-scoring step was applied; titles and abstracts were screened independently by two reviewers (Section 2.2), and records found in more than one source were reconciled informally. Selection was purposive and synthesis interpretive; no systematic reproducibility is implied.

#### 2.1.3 Authorship and clinical perspective

The authors are computer scientists, and the manuscript has no clinical co-author. The D1–D4 deployment classes (Section 5.3), the severity judgments in Table 2 and Figure 2, and the statements about clinical workflow and the feasibility of the research agenda constitute the authors’ analytic framing, derived from the published clinical and informatics literature and from practice guidance such as the ADA Standards of Care (American Diabetes Association Professional Practice Committee for Diabetes, 2026). These elements were not validated with practicing endocrinologists, primary care physicians, or diabetes educators and should be read as proposals for clinical review, not as clinically validated findings (Section 9). No clinical recommendation should be drawn from them. Structured review by endocrinologists, ophthalmologists, and diabetes educators is the intended next step.

**Table 2:** The era-III safety matrix for diabetes AI: failure class × mechanism × current mitigation × evidence status.

Failure class	Mechanism	Most-affected deployment class (D1–D4)	Current mitigation	Mitigation evidence status
Glycemic-critical hallucination	Fluent generation under sparse/conflicting training signal	D3–D4	RAG; tool-constrained decoding; refusal policies	Partial, retrospective only; no L1 identified
Automation bias	Over-trust of fluent output; vigilance decay	D2 (also D1)	Override telemetry; confidence display; and forced friction.	Instrument exists; not, to our knowledge, applied in endocrinology
Corpus-inherited inequity	Pretraining-scale demographic/device skew	All classes	Subgroup evaluation; targeted adaptation	Normative (Section 6.3); rarely practiced
Feedback-ratchet inequity	Deployment → data → representation loop	All classes	None identified	Unmeasured; conceptual risk
Adaptation privacy leakage	Memorization + extraction from fine-tuned models	D1–D3	DP fine-tuning; de-identification; federated adaptation	Techniques exist; no diabetes-specific audits identified
Synthetic-data privacy	Memorization in generative models; DP, de-identification, and audits conflated or unreported	Research infrastructure	DP-SGD; memorization audits	Rarely applied in diabetes generative pipelines

**Figure 2:**
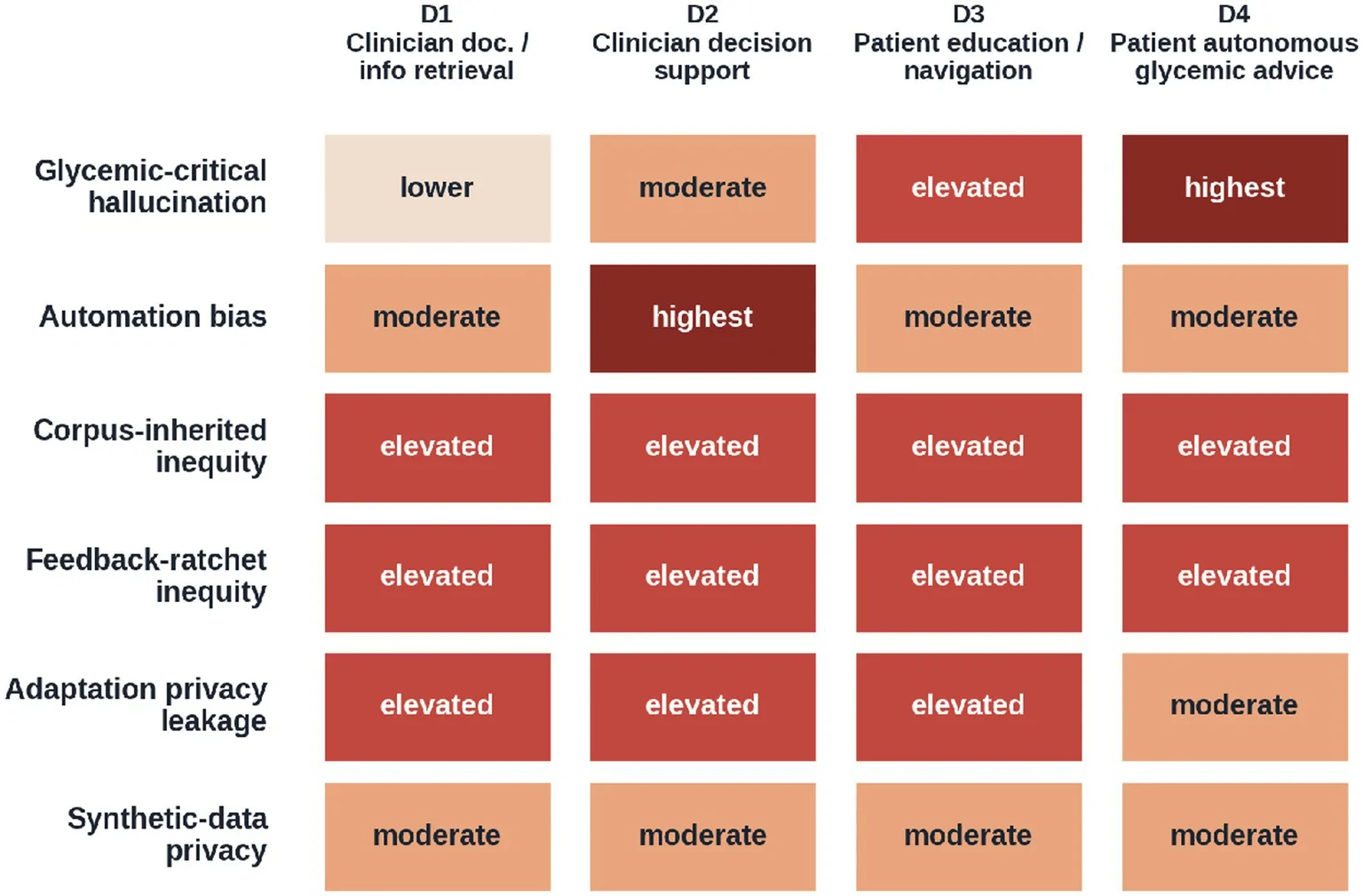
Safety matrix heat map. Six failure classes (rows, from Table 2) by deployment classes D1–D4 (columns), shaded by risk severity; risk concentrates in D3–D4. Source: Authors’ qualitative severity judgments (Section 7), not measured incidence and not clinically validated (Section 2.1).

### 2.2 Literature identification and scope

Literature was identified through purposive searches of open bibliographic sources (IEEE Xplore, PubMed/MEDLINE, arXiv, Europe PMC, and OpenAlex) conducted in August, 2026. The searches were restricted to reports published from January 2016 to December 2025 (Section 2.1) and to English-language sources, focusing on three themes: diabetes and its complications; FMs and generative-AI methods (including large language models and vision-language models); and clinical application and evaluation (screening, diagnosis, prediction, management, reliability, attention, interpretability, and explainability). The searches were purposive and not exhaustive.

#### 2.2.1 Search strings

The three themes were combined with AND. Theme 1 (diabetes and complications): (“diabetes mellitus” OR “type 1 diabetes” OR “type 2 diabetes” OR “diabetic retinopathy” OR “diabetic kidney disease” OR “continuous glucose monitoring”). Theme 2 (FMs and generative AI): “foundation model*” OR “large language model*” OR “self-supervised learning” OR “masked autoencoder” OR “generative AI”). Theme 3 (clinical application and evaluation): (“screening” OR “diagnosis” OR “prediction” OR “evaluation” OR “validation” OR “calibration” OR “safety”). Syntax was adapted to each database.

#### 2.2.2 Screening process

Titles and abstracts retrieved from the database searches were screened independently by two reviewers against predefined eligibility criteria (below). Disagreements were resolved through consensus discussion, followed by full-text review of potentially eligible reports. Agreement statistics were not calculated, and no counts of records are reported (Section 2.1). Trial registrations were also checked on ClinicalTrials.gov in August, 2026 (Section 6.1), outside the literature window of Section 2.1, and reported there as time-sensitive. Scopus, Web of Science, DBLP, ACM Digital Library, and Google Scholar were not searched (Section 9). The corpus is therefore a purposive sample, and the principal studies on which the conclusions rest are summarized descriptively in Table 3 (Section 6.2). Selection bias remains possible: visible, English-language, open-access, and highly cited work may be over-represented, and null or negative results under-represented.

**Table 3:** Descriptive summary of the principal studies discussed in Sections 4–6: model or class, design and data, validation type, evidence level, and note.

Study	Model or class	Design and data (as discussed)	Validation type	Level	Note
Gulshan et al. (2016)	Task-specific CNN (Inception-v3); era II	DR grading from fundus photographs; AUROC ≈0.98 (Table 8)	Internal and external sites, as graded in Table 8	L2–L3	Era-II reference point; label-hungry, single task
Abràmoff et al. (2018)	IDx-DR autonomous DR detection; era II	Pivotal prospective trial supporting FDA *De Novo* authorization (2018)	Prospective, real workflow	L1	Only domain where the full ladder was climbed; task-specific, not an FM
Zhou et al. (2023)	RETFound; ViT-L (~300 M), masked autoencoding	1.6 M retinal images (UK Biobank, Moorfields); adapted to DR grading (EyePACS, APTOS, Messidor-derived), sight-threatening disease (Moorfields), MI, heart failure, and stroke prediction	External-site (retinal tasks); internal held-out UK Biobank (systemic)	L2/L3	Pretraining–evaluation cohort overlap; systemic AUROC 0.58–0.64; developer-reported; L2 = retinal tasks, L3 = systemic
Yew et al. (2025)	Independent comparison: RETFound vs. traditional deep learning	Ocular and systemic disease detection (arXiv preprint)	Comparative evaluation	Not graded	ImageNet-pretrained networks are largely comparable when labels are plentiful.
Isztl et al. (2025)	Independent comparison across retinal imaging tasks	Evaluation of when domain-specific FMs justify their cost (arXiv preprint)	Comparative evaluation	Not graded	FM advantage narrows or disappears when labels are ample.
Zhang et al. (2024)	RETFound	Community screening evaluation including decision-curve analysis	External population (community screening)	L2	One of the few retinal FM reports with decision-curve analysis
Nie et al. (2023), Zerveas et al. (2021)	Patch-based and masked time-series transformers	General time-series pretraining adapted to CGM forecasting and hypoglycemia anticipation on public datasets	Benchmark: cross-dataset zero-shot	L4	Not shown to translate into clinical-alert performance
Marling and Bunescu (2020)	OhioT1DM dataset; LSTM/GRU forecasters; era II	Single-study T1DM cohorts of tens to hundreds of patients	Internal held-out	L3	Brittle cross-dataset transfer; T1DM-centric
Rasmy et al. (2021), Li et al. (2020)	Med-BERT; BEHRT (patient-sequence FMs)	Masked-language objective on structured records from millions of patients; adaptable to diabetes endpoints	Internal held-out benchmark (Table 6)	L3–L4	Coding-artifact leakage; rare-event sample efficiency
Singhal et al. (2023a, b)	Med-PaLM; Med-PaLM 2	MedQA 67.6 and 86.5%; physician-graded long-form answers on MultiMedQA	Benchmark	L4	Contamination is plausible; it is not diabetes-specific; the exam score is an upper bound.
Nori et al. (2023), OpenAI (2023)	GPT-4 family (closed weights)	Medical licensing-style benchmarks, including diabetes content	Benchmark	L4	Closed model; corpus undisclosed; preprint or technical report
NCT07350486 (registry record)	LLM-enabled coordinated-management tool	Cluster-randomized trial for hypertension, diabetes, and dyslipidemia; not yet recruiting at last check (August, 2026)	Prospective (planned)	No results	Planned L1 design; no outcome data; outside the literature window

Evidence levels (L1–L4) follow Section 2.3 and are the authors’ interpretive grading, not a quality rating or a formal risk-of-bias assessment. The table is a descriptive summary of studies discussed in the text, not an exhaustive or systematically selected list. “Not graded” marks comparative or non-diabetes-specific reports.

#### 2.2.3 Material considered

We considered (i) studies pretraining or adapting large-scale models on diabetes-relevant data; (ii) reports of external, prospective, or regulatory evaluation of clinical AI relevant to diabetes; (iii) methodological and position papers defining the FM, evaluation, or safety frameworks we critique; and (iv) dataset and benchmark papers. Diabetes or a diabetic complication had to be a task, population, or evaluation target. Only English-language reports were considered, because the corpora, benchmarks, and clinical-LLM evaluations under review are overwhelmingly in English (Section 9). The main sources were peer-reviewed articles, full-length conference papers from the relevant machine-learning and medical-imaging venues, and regulatory documents (for example, FDA clearance summaries and *De Novo* decisions); preprints were used with caution and are identified where they are the primary source for a claim, for example, the GPT-4 technical report (OpenAI 2023) for a closed model. Editorials, commentaries without original analysis, conference abstracts without a full text, theses, and retracted articles were not used.

### 2.3 Evidence grading

Evidence grading: Each empirical claim discussed in this review is assigned one of four evidence levels, used consistently in Tables 3, 4–7:

- L1: prospective or randomized clinical evaluation in a real workflow;
- L2: external validation on independent, geographically or temporally distinct data;
- L3: internal validation on held-out data from the source cohort;
- L4: benchmark-only evaluation (public datasets; no clinical provenance of test data).

**Table 4:** Ophthalmic FMs and retinal self-supervised models relevant to diabetes: architecture, objective, and evidence level.

Model/family	Backbone & scale	Pretraining corpus & objective	Diabetes-relevant downstream tasks	Best evidence level	Reported headline metric	Key limitation
RETFound	ViT-L (~300 M)	1.6 M retinal images (UKBB + Moorfields); MAE	DR grading; sight-threatening disease; MI/HF/stroke prediction	L2 (retinal tasks); L3 (systemic)	DR grading AUROC ≈0.94 (APTOS-2019); 0.82–0.88 on IDRiD/MESSIDOR-2; systemic AUROC 0.58–0.64	Pretraining–evaluation cohort overlap; systemic performance subclinical
ImageNet-init. ViT fine-tune (era-II/III hybrid baseline)	ViT-B/L	ImageNet-21 k; supervised	DR grading on EyePACS/APTOS	L4	AUROC ≈0.93–0.97	Domain-foreign pretraining; serves as FM baseline
Contrastive retinal SSL (SimCLR/DINO variants on fundus)	ResNet/ViT variants	10^5^–10^6^ fundus images; contrastive	DR grading, vessel segmentation	L3–L4	Comparable to supervised at 1–10% labels	Corpus fragmentation; no unified checkpoint ecosystem
OCT-domain FMs (self-supervised on OCT/OCT-A volumes)	ViT/hybrid CNN-transformer	10^5^–10^6^ OCT B-scans; MAE/contrastive	DME detection; drusen/IRM segmentation (diabetes-adjacent)	L3–L4	Task-dependent	Diabetes-specific evaluation sparse; OCT-A in DKD underexplored
Multimodal retinal-text FMs (fundus-image + report alignment)	ViT + LLM encoder	Image–caption pairs; contrastive/next-token	Automated retinal reporting; DR grading	L4	Emerging	Report availability biased to tertiary centers

Evidence levels per Section 2.3: L1 prospective, L2 external, L3 internal, L4 benchmark-only. Reported metrics are drawn from the primary sources cited in each row; given rapid iteration in this literature, readers should consult those sources directly for the most current values. Cross-table comparison caveat: heterogeneity in labeling protocol (grade vs. referral decision), prevalence, and preprocessing makes cross-row metric comparisons qualitative; row order reflects maturity, not merit (plotted in figure 6).

**Table 5:** Foundation-model approaches for CGM time series in diabetes.

Approach	Representative objective	Typical backbone	Pretraining data scale	Diabetes downstream tasks	Evidence level	Distinct failure mode
Generic time-series FM → CGM	Masked patch reconstruction; forecasting-as-pretraining	Patch transformer (PatchTST-lineage); TS-MAE	10^6^–10^9^ generic series + CGM adaptation	Glucose forecasting (30–120 min); hypo-anticipation	L4 (zero-shot cross-dataset emerging)	Sampling-rate and calibration-level mismatch across CGM devices
Diabetes-native CGM encoder	Masked reconstruction + next-window prediction on CGM only	Temporal transformer, 10^7^–10^8^ params	10^3^–10^5^ patient-years of CGM	Hypoglycemia prediction; variability phenotyping; meal/exercise encoding	L3–L4; no L1 identified	Proprietary-corpus opacity; no public reproduction
Generative synthetic CGM	Diffusion/autoregressive generation	Diffusion U-Net; causal transformer	Same corpora as above	Data augmentation; predictor stress-testing; privacy-preserving sharing	L4 (validity uncertified)	Artifact propagation into downstream clinical models
Multimodal CGM + wearables+bolus (T1DM-centric)	Contrastive alignment across sensor streams	Cross-modal transformer	10^2^–10^4^ patient-years	Excursion anticipation; insulin-on-board inference	L3–L4	T2DM transfer largely untested

**Table 6:** EHR and clinical-text FMs: diabetes adaptation surface.

Model class	Pretraining objective	Diabetes-relevant adaptations	Evidence level	Critical open issue
Patient-sequence FMs (structured events)	Next-visit/masked-value prediction	Incident DKD: hypo-risk on intensification; complication-trajectory forecasting	L3–L4	Coding-artifact leakage; rare-event sample efficiency
Clinical-note LLM encoders	Masked-language/contrastive notes	CKD stage extraction, DFU-wound description parsing, and guideline-concordance audits	L3–L4	Institutional note-style shift
Instruction-tuned clinical LLMs (Med-PaLM 2, GPT-4-class; cf. Ouyang et al., 2022)	Supervised fine-tuning (SFT) + reinforcement learning from human feedback (RLHF) on medical corpora	QA, documentation, patient education (Section 5)	L4 benchmarks; L1 only for consumption studies	Benchmark contamination; hallucination
Multimodal EHR FMs (structured + text + imaging)	Cross-modal alignment	Unified diabetes-complication risk from full record	L4	No diabetes external validation identified
EHR generative models	Autoregressive record generation	Synthetic cohorts for DKD/hypo studies	L4	Privacy guarantees; downstream validity uncertified

**Table 7:** LLM adaptation strategies for diabetes care: mechanism, capability, and verifiability trade-offs.

Layer	Mechanism	Diabetes deployment surface	Capability retained	Verifiability gained	Documented failure mode
Prompting/CoT	In-context specification	Education content; triage-style QA	Full	None (output varies by phrasing)	Prompt injection; inconsistent refusal behavior
RAG	Retrieval-conditioned generation	Guideline-grounded QA; formulary-aware advice	Near-full, plus currency	Provenance of retrieved chunks; citation checks	Retrieval failure → confident ungrounded answer; stale corpus
PEFT fine-tuning	Low-rank weight updates on domain corpora	Tone/register matching; local protocol conformance	Domain-tuned	Training-data audit (in principle)	Bias amplification; catastrophic forgetting of safety behavior
Agentic tool use	The planner invokes deterministic tools.	Dose-checking; interaction screening; CGM interpretation	Task decomposition	Hard constraints enforced by non-neural components	The planner selects the wrong tool; error propagation across calls
Fully autonomous advice	Unconstrained generation to patients	(Proposed commercial chatbots)	Full	None	Hallucinated dosing (Section 7.1): no L1 evidence identified

#### 2.3.1 Types of external validation

Level L2 groups three distinct kinds of external evidence, which this review keeps apart (Section 6.3):

- External-site validation tests a frozen model on data from a different institution, device, camera, or acquisition workflow, but typically from a similar patient population and the same calendar period as development. It addresses site, scanner, and workflow shift; it does not address changes in who is tested or when.
- External-population validation tests the model in a different demographic, geographic, or clinical setting, whatever the site: for example, another country or ethnic mix, community screening versus tertiary referral, T2DM versus T1DM, a different age band, or pregnancy.
- Temporal (“era”) validation tests the model on data collected after the development data, from the same or another source, so that changes in practice, sensor or camera generation, coding conventions, and treatment era are exposed.

Validation on an external population and era therefore means a different population and a later period together, and it makes a stronger claim than validation at an external site. For example, a retinal model tested on another hospital’s camera has passed external-site validation but not external-population or temporal validation if that hospital serves a similar referred adult population in the same years; a CGM model tested on a different sensor brand in the same clinic is likewise an external-site (device) test only. A model that passes one kind is graded L2 but is not thereby shown to transport across the others, and Section 6.3 asks for each kind to be reported separately.

This grading is central to the review’s argument: Section 6 uses it to show, qualitatively, that the era-III diabetes literature is heavily skewed toward L3–L4. It is an interpretive device applied by the authors based on what each report states, not a validated instrument, and no inter-rater reliability statistics are reported.

#### 2.3.2 Risk of bias

The L1–L4 scheme grades the level of validation, not the internal validity of individual studies, and should not be read as a quality rating. Formal instruments (PROBAST, Wolff et al., 2019; QUADAS-2, Whiting et al., 2011; APPRAISE-AI, Kwong et al., 2023) were not applied for three reasons: this is a narrative review that did not systematically enumerate or screen studies (Section 2.1), so study-by-study appraisal would imply a completeness it does not claim; the literature reviewed is dominated by pretraining, benchmark, and preprint reports to which these tools map poorly; and the aim is to characterize the validation ladder, not to rate studies or pool effect estimates. Risk of bias was instead considered qualitatively and reported alongside the evidence: validation level, overlap between pretraining and evaluation cohorts, benchmark contamination, closed-model opacity, preprint status, and whether calibration, subgroup (fairness) analysis, and external or prospective evaluation were reported (Sections 4–6; Tables 4–7). These are the authors’ unreplicated judgments and a critical appraisal, not a formal risk-of-bias assessment. Applying PROBAST, QUADAS-2, or APPRAISE-AI to the L1–L2 studies, where such tools fit best, is a priority for follow-up systematic work.

#### 2.3.3 Why each risk-of-bias instrument was not applied

PROBAST (Wolff et al., 2019) assesses risk of bias and applicability in studies that develop, validate, or update a multivariable prediction model, using participant, predictor, outcome, and analysis domains. Most era-III reports reviewed here are pretraining or adaptation papers in which one backbone serves many tasks, the pretraining corpus does not define a participant cohort, and there is no single predictor–outcome pair, so the domains cannot be completed in a meaningful way. PROBAST would fit only some downstream task-specific prediction studies (for example, systemic-disease prediction from retinal images), and rating those alone would misrepresent the literature. QUADAS-2 (Whiting et al., 2011) assesses diagnostic accuracy studies and requires an index test, a reference standard, and patient flow and timing. It applies only to a minority of retinal grading studies, but not to CGM forecasting, EHR sequence models, LLM question-answering benchmarks, or synthetic-data studies, none of which have a diagnostic reference standard. APPRAISE-AI (Kwong et al., 2023) scores clinical AI studies across domains such as clinical relevance, data quality, methodological conduct, robustness, reporting, and reproducibility. For closed-weight models and pretraining reports whose corpus and model details are undisclosed, the data-quality and reproducibility domains would mostly record non-disclosure so that a score would describe transparency rather than study quality; the opacity itself is treated here as a finding (Sections 2.4 and 6.1). Where the tools do fit, namely the few L1–L2 studies such as the IDx-DR pivotal trial and the external-site retinal evaluations, the relevant bias signals (cohort overlap, external-validation type, calibration and subgroup reporting, preprint status) are noted in Table 3, and formal application remains a priority for follow-up systematic work.

### 2.4 Analytic approach and synthesis

Studies were analyzed interpretively. For each model or study discussed, we considered architecture class, parameter scale (where disclosed), pretraining corpus size, modality, and provenance; pretraining objective; adaptation strategy; downstream task(s); reported primary metric(s); validation level (L1–L4); calibration reporting; fairness subgroup analysis; and regulatory status. These considerations served as an analytic frame for Tables 3, 4–7 not as a formal data-extraction form. Where parameter counts or corpus sizes are undisclosed (typical for closed-weight models), this is noted as “undisclosed” and treated as a governance finding, not a footnote.

Synthesis was narrative and critical. Within each modality, we contrasted what was claimed with the evidence level and the calibration, fairness, and regulatory reporting available, organized by the three-era taxonomy (Section 3), the L1–L4 evidence levels (Section 2.3), and the safety and equity domains (D1–D4). Because of the heterogeneity of tasks, metrics, and populations, no effect estimates were pooled, and no meta-analysis was attempted; cross-study metric comparisons are qualitative. Absence claims were made only after targeted checking of the literature we reviewed for the specific evidence type in question.

## 3 A three-era taxonomy of AI in diabetes care

### 3.1 The taxonomy

We organize the field’s development into three eras distinguished by where the knowledge in the model comes from (Table 1; timeline in Figure 3; captions and sources per Bommasani et al., 2021; Gulshan et al., 2016; Abràmoff et al., 2018; Zhou et al., 2023).

**Figure 3:**
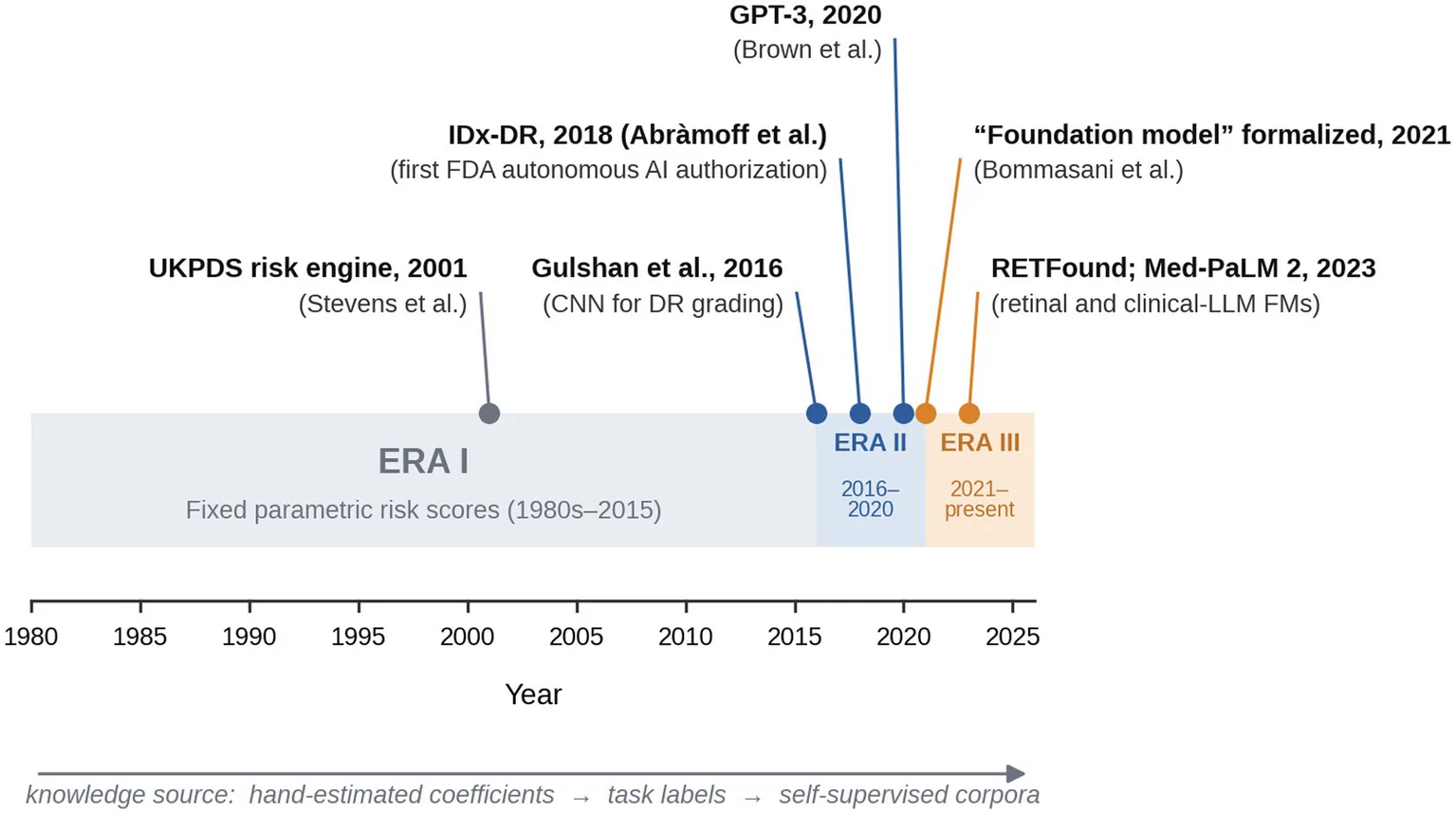
Three-era taxonomy timeline (1980–2025). Era I, era II (2016–2020), and era III (2021–present) are shown as horizontal bands with landmark artifacts (UKPDS risk engine, 2001; Gulshan et al., 2016; IDx-DR, 2018; GPT-3, 2020; formalization of “FM,” 2021; RETFound and Med-PaLM 2, 2023), annotated with the shift in knowledge source from coefficients to labels to self-supervised corpora. Source: Authors’ conceptual illustration based on Stevens et al. (2001), Gulshan et al. (2016), Abràmoff et al. (2018), Brown et al. (2020), Bommasani et al. (2021), and Zhou et al. (2023); era boundaries are conventions (Section 3.1); not empirical data.

Era I: Fixed parametric risk scores (1980s–2015): Knowledge is injected by human estimation of a small number of coefficients over hand-engineered features (age, HbA1c, blood pressure, duration). Examples: the UKPDS risk engine (Stevens et al., 2001); DCCT retinopathy progression models (Diabetes Control and Complications Trial Research Group, 1993); ACR-based diabetic kidney disease (DKD) staging per KDIGO criteria (Kidney Disease: Improving Global Outcomes (KDIGO) Diabetes Work Group, 2022). Properties: fully auditable, cheap, but limited by feature expressivity and unable to represent nonlinear interactions or imaging/temporal signals.

Era II: Task-specific supervised deep learning (2016–2020): Knowledge is learned end-to-end from labeled task data. Characteristic architectures: CNNs (Inception-v3 in Gulshan et al., 2016); EfficientNets in subsequent, purpose-built CNNs for diabetic foot ulcer classification (Goyal et al., 2020), a complication that remains a leading precursor of amputation (Armstrong et al., 2017), LSTMs/GRUs for CGM forecasting, and XGBoost for tabular DKD risk. Properties: superhuman performance on narrow benchmarks (DR grading AUROC >0.95 across multiple studies) but (i) label-hungry, (ii) task-brittle (a DR grader cannot predict anti-VEGF response), (iii) distributionally fragile; performance degrades sharply under camera, site, and demographic shift, as demonstrated systematically in the external-validation literature (e.g., Beede et al., 2020).

Era III: Foundation models and pretrained generalists (2021–present): Knowledge is acquired through self-supervised pretraining on broad corpora and adapted to tasks with few labels or no labels (in-context). Subclasses: (a) modality FMs (RETFound for retina; physiological time-series transformers for CGM); (b) clinical LLMs (Med-PaLM 2, GPT-4, and successors) covering diabetes text tasks; (c) multimodal FMs aligning imaging, temporal, and text modalities; (d) generative data models producing synthetic CGM/EHR.

#### 3.1.1 Why era III starts in 2021

The eras are defined by the source of a model’s knowledge, so the start date marks when large-scale self-supervised pretraining became a standard approach to building clinical models, not when the first such model appeared. We use 2021 for three reasons. First, the paradigm was named and formalized in 2021 (Bommasani et al., 2021). Second, the enabling methods for the modalities in this review matured around that time: in-context learning at scale just before (GPT-3; Brown et al., 2020) and, in 2021–2022, self-supervised vision transformers (Caron et al., 2021; He et al., 2022), transformer pretraining on patient sequences (Rasmy et al., 2021), and transformer representation learning for multivariate time series (Zerveas et al., 2021). Third, it leaves era II (2016–2020) as the period when task-specific deep learning was both the research frontier and the source of the first regulatory authorization (IDx-DR, 2018). The date is a convention. Era-II-style models continue to be built and deployed after 2020, and the best-known retinal and clinical-LLM FMs relevant to diabetes care (RETFound, Med-PaLM 2) appear only in 2023. A 2023 start would therefore change little in the diabetes literature itself, whereas a 2020 start would admit general-domain models with no diabetes-specific adaptation. Where a conclusion depends on the date, we say so.

### 3.2 What “foundation” changes for each diabetes modality

The taxonomy is modality-generic; its clinical consequences are modality-specific (Table 8). The unifying pattern: in every modality, era III replaces supervised endpoint learning with representation learning of the patient signal itself, which retrofits the same backbone to previously unlinked endpoints: retinal FMs predicting cardiovascular events, CGM FMs transferring across T1DM/T2DM, and EHR FMs supporting both risk prediction and clinical documentation (the adaptation stack is diagrammed in Figure 4).

**Table 8:** Era-II → era-III transition by diabetes data modality (summary; detailed in Sections 4–5).

Modality	Era-II exemplar (evidence level)	Era-III exemplar (evidence level)	What changes technically	Key unresolved question
Retinal imaging	Inception-v3 DR grader, AUROC ~0.98 (L2–L3)	RETFound, ViT-L, and MAE on 1.6 M images (L2 on 3 retinal tasks; L3 systemic prediction)	CNN, ImageNet transfer → domain-native masked pretraining; grading → prognosis + systemic endpoints	Do retinal FMs retain calibration for ocular disease in non-referred populations?
CGM time series	LSTM/GRU glucose forecasters (L3)	Pretrained CGM/physiological transformers (L3–L4)	Per-patient sequence models → shared temporal representations; forecast → variability, hypo-risk, behavioral encoding	Inter-device and inter-regime (T1DM vs. T2DM) transferability
Tabular EHR	XGBoost DKD risk models (L2–L3)	Clinical EHR FMs adapted to diabetes endpoints (L3–L4)	Feature engineering → self-supervised token/visit modeling	Rare-event (end-stage kidney disease, ESKD) learning; leakage from coding artifacts
Clinical text/patient interaction	Rule-based chatbots, scripted education	Med-PaLM 2, GPT-4-class models on diabetes QA (L4; L1 for consumption trials only)	N/A (new capability class)	Safety of autonomous patient-facing dosing/diet advice
Synthetic data	SMOTE-style augmentation (L4)	Diffusion/GPT-style synthetic CGM & EHR (L4)	Interpolation → generative realism	Privacy guarantees; downstream validity of synthetic-trained models

**Figure 4:**
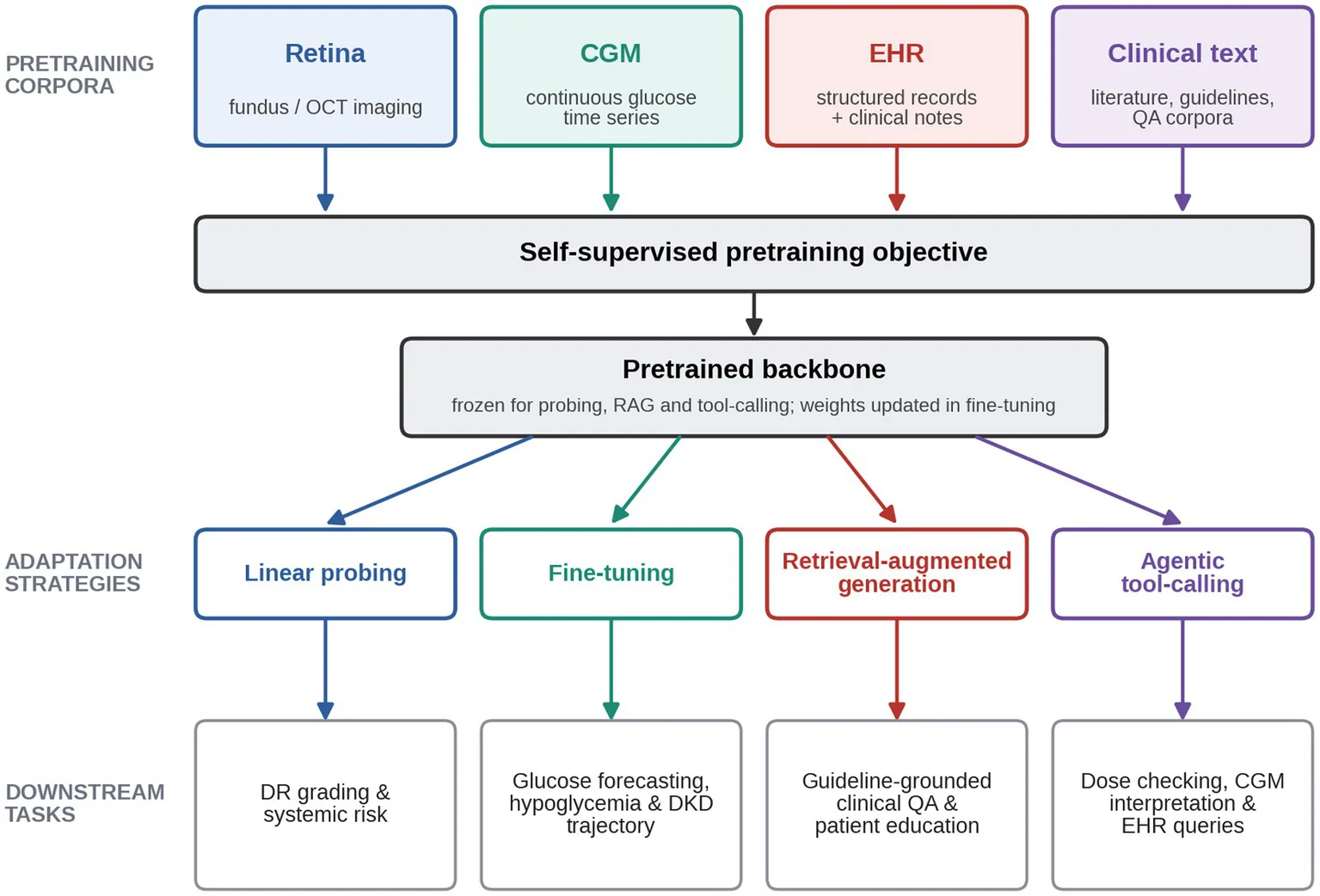
Foundation-model adaptation stack. Pretraining corpora (retina, CGM, EHR, clinical text) feed a self-supervised pretraining objective and a frozen backbone, which supports four parallel adaptation strategies (linear probing, fine-tuning, retrieval-augmented generation, and agentic tool-calling) and their downstream diabetes tasks. Source: Authors’ conceptual diagram; no data.

### 3.3 Roadmap

Section 4 analyzes modality-level FMs (retina, CGM, EHR/multimodal) with architecture- and objective-level technical detail and Tables 4–6. Section 5 examines clinical LLMs for diabetes: capability benchmarks, adaptation strategies (RAG, agentic workflows), and the diabetes-specific deployment surface. Section 6 presents the evaluation crisis and critically appraises evidence levels. Section 7 analyzes safety, fairness, and privacy as constraints that determine deployability. Section 8 sets out the research agenda; Sections 9–11 give limitations, discussion, and conclusions.

## 4 The foundation-model landscape by data modality

This section analyzes the era-III landscape modality by modality. For each modality, we follow the same analytical sequence: pretraining corpus and objective, architecture, adaptation strategies, downstream diabetes tasks and reported performance, and (critically) the validation level attained, because Section 6 will show that the gap between capability and validated capability is the field’s defining pathology (mapped in Figure 5).

**Figure 5:**
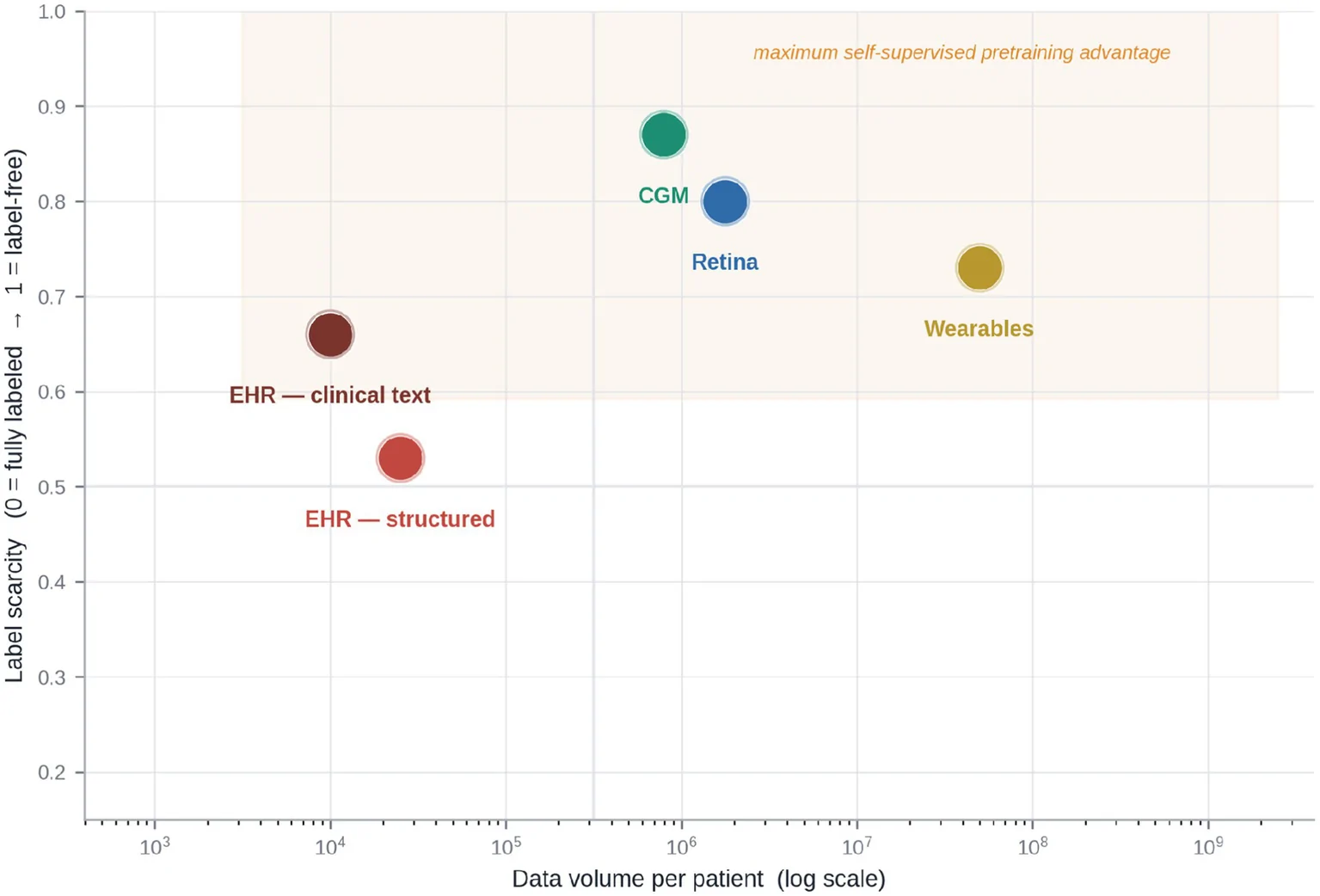
Modality map of diabetes data. Data volume per patient (log scale, *x*-axis) against label scarcity (*y*-axis) for CGM, retina, EHR (structured and clinical text), and wearables; shading marks where self-supervised pretraining is expected to have the greatest advantage. Source: Authors’ qualitative judgments; positions are not measured values.

### 4.1 Ophthalmic foundation models: the retina as a diabetes window

#### 4.1.1 Why the retina is the most advanced FM frontier for diabetes

The retina occupies a unique position in the diabetes-AI landscape for reasons that are anatomical, economic, and computational. Anatomically, DR is a microvascular disease visible *in situ*: the same microangiopathic processes that damage retinal capillaries damage renal glomeruli and, to a documented extent, coronary and cerebral vasculature (Cheung and Wong, 2008), making the retinal photograph a non-invasive window onto systemic microvascular health. Economically, fundus photography is cheap, non-invasive, protocolized, and already deployed at a population scale in screening programs (UK, Singapore, India), yielding millions of archived images. Computationally, these archives are exactly the kind of large, homogeneous-modality, weakly-labeled corpus that self-supervised pretraining requires. The result: the retina is, to our knowledge, among the first organs for which a genuinely domain-native FM exists (cf. Keane and Topol, 2018).

#### 4.1.2 RETFound: architecture, objective, and reported capabilities

RETFound (Zhou et al., 2023) is the paradigmatic case and merits technical unpacking:

- Backbone: Vision Transformer, large configuration (ViT-L; ~300 M parameters), 16 × 16 patch tokenization of 224 × 224 crops: the standard ViT recipe (Dosovitskiy et al., 2021) rather than a novel architecture. This is itself a finding: the ophthalmic FM frontier is running on generic architectures, with domain specificity supplied entirely by the pretraining corpus.
- Pretraining objective: masked autoencoding (MAE; He et al., 2022): random masking of ~75% of image patches and reconstruction of the missing patches in pixel space. The choice is consequential: unlike contrastive objectives (SimCLR/DINO-style; Chen et al., 2020; Caron et al., 2021), MAE learns dense, local texture representations, which plausibly suit microvascular pathology (microaneurysms, hemorrhages, and neovascularization are small, dense-texture anomalies) better than global-scene objectives. This hypothesis (that MAE-style objectives are preferentially suited to microvascular imaging) is testable and, to our knowledge, not yet explicitly tested; it is a concrete research gap we return to in Section 8.
- Corpus: 1.6 million retinal images from UK Biobank and Moorfields Eye Hospital, spanning ethnicities and both white-light and confocal-scanning-laser ophthalmoscope modalities, but predominantly adult UK Biobank participants, a point of consequence for the generalization analysis below.
- Adaptation and reported results: linear probing and fine-tuning to (i) DR severity grading [internal validation on EyePACS (Cuadros and Bresnick, 2009), APTOS, and Messidor-derived benchmarks (Decencière et al., 2014)], (ii) sight-threatening retinal disease detection at Moorfields (external-site validation relative to pretraining data), and (iii) systemic disease prediction (incident myocardial infarction, heart failure, and stroke from retinal images) where RETFound improved over ImageNet-initialized ViTs and task-specific CNNs. Performance gains over task-specific baselines were consistent but moderate (reported AUROC improvements in the ~1–4-point range depending on task).

#### 4.1.3 What RETFound does and does not establish

The most-quoted capability claim (that retinal images predict systemic disease) rests on the weakest evidence in the paper’s own grading: systemic-prediction tasks were validated on UK Biobank held-out data (L3 in our taxonomy), with the pretraining corpus drawn from the same biobank, and the reported AUROCs for cardiac endpoints (~0.58–0.64, disease-specific) (Figure 6) are far below clinical deployment thresholds and below established non-imaging risk scores. Two failure modes of the era-II evaluation culture therefore persist into era III in attenuated form: (i) domain-cohort entanglement: pretraining, adaptation, and evaluation populations overlap; (ii) metric inflation by framing: relative AUROC improvements over baselines are reported against absolute performance that is clinically modest. Neither is a flaw unique to RETFound; both are systemic (Section 6). What RETFound’s developers report, and what independent comparisons partly corroborate, is a label-efficiency advantage: with a few hundred labels per task, the pretrained backbone is reported to match or exceed fully supervised models trained on many more task labels. Independent head-to-head comparisons find this advantage clearest when labels are scarce, and it narrows or disappears when labels are ample (Yew et al., 2025; Isztl et al., 2025).

**Figure 6:**
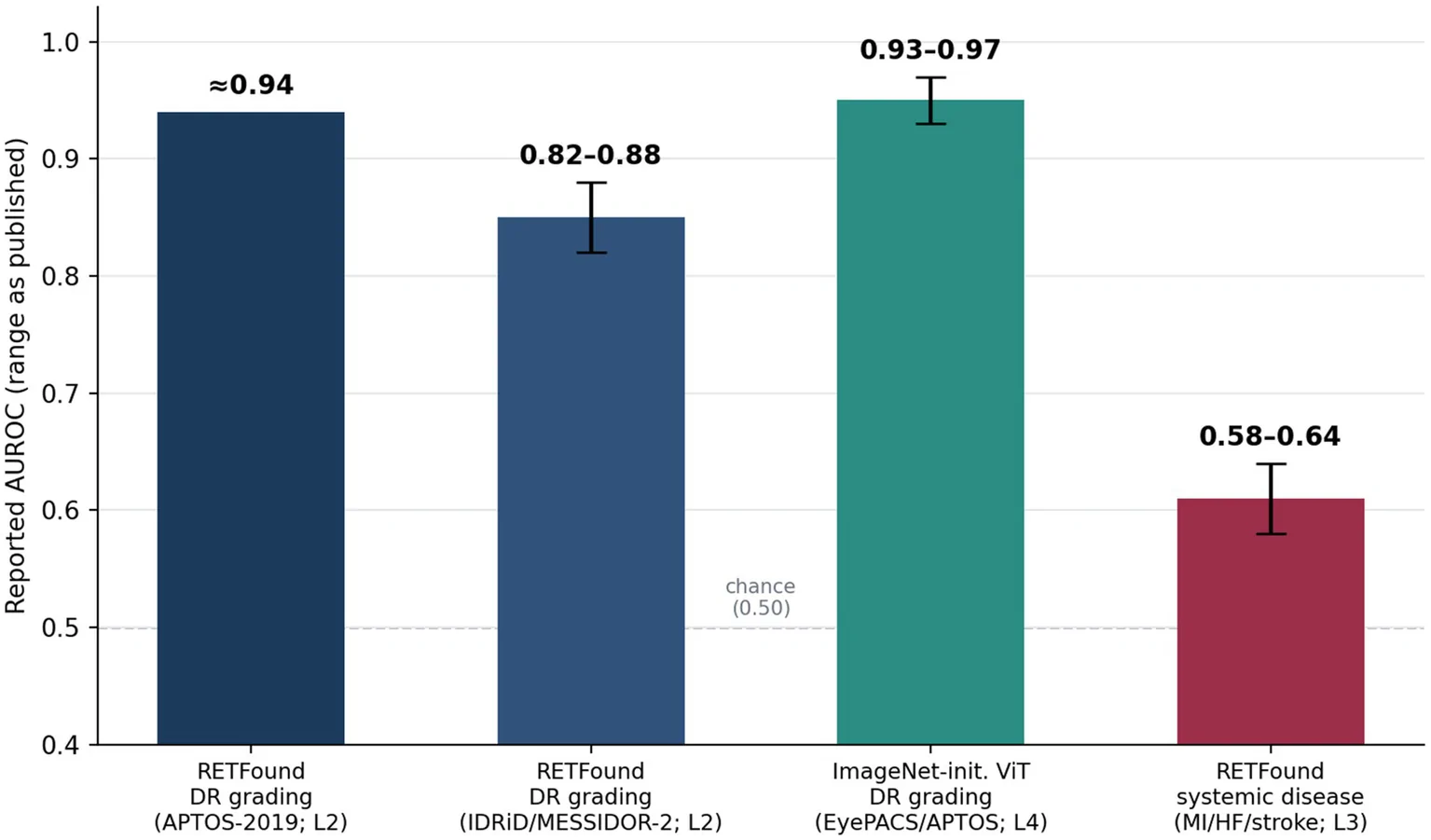
Reported AUROC by ophthalmic foundation-model task (Table 4). RETFound DR grading (≈0.94 on APTOS-2019; 0.82–0.88 on IDRiD/MESSIDOR-2; L2), ImageNet-initialized ViT DR grading (0.93–0.97, L4), and RETFound systemic-disease prediction from retinal images (MI, heart failure, stroke; 0.58–0.64; L3). Bars mark the midpoint of each reported range, and error bars its full extent; the dashed line marks chance (AUROC = 0.50). Cross-row comparison is qualitative because labeling protocols and cohorts differ (Section 4.1.3). The first bar (~0.94) is a single reported value and has no error bar. Source: Values reported in the original studies listed in Table 4, compiled and redrawn by the authors.

#### 4.1.4 The open retinal questions for diabetes

Three questions specific to diabetes remain open. First, generalization to the screening margin: RETFound’s pretraining corpus is dominated by the UK Biobank volunteers: healthier and ethnically narrower than the populations where DR burden concentrates (South Asia, Sub-Saharan Africa; Teo et al., 2021); whether retinal FM representations transfer to field-grade, unmydriatic, higher-humidity photography in those settings is, to our knowledge, untested (cf. Bellemo et al., 2019; Ting et al., 2017). Second, calibration for triage decisions: DR screening is a referral-threshold problem where the clinically relevant metric is sensitivity at fixed specificity (and the cost asymmetry of missed proliferative disease); we identified few retinal FM reports with calibration curves or decision-curve analysis across sites (a community screening evaluation of RETFound that included decision-curve analysis is a notable exception; Zhang et al., 2024). Third, the renal extension: if retinal vasculature encodes systemic microvascular state, the highest-value diabetes target is not MI prediction but diabetic kidney disease trajectory prediction from retinal images: a task for which era-II evidence exists in nascent form but for which, to our knowledge, no FM has yet been adapted and validated (an “L0” gap: the task is absent from the literature we reviewed).

##### 4.1.4.1 Beyond fundus photography

Fundus photography is not the only retinal modality relevant to diabetes. Optical coherence tomography (OCT) and OCT angiography (OCT-A) are central to diabetic macular edema management and to capillary-level microvascular assessment, and OCT-domain FMs appear in Table 4. Evidence for them in diabetes is thinner than for fundus models: reported evaluations are largely L3–L4, diabetes-specific validation is sparse, and OCT-A in diabetic kidney disease is underexplored. Conclusions drawn in this section from RETFound-style fundus models should not be assumed to transfer to OCT.

### 4.2 Continuous glucose monitoring: foundation models for physiological time series

#### 4.2.1 The modality’s structure and why it invites pretraining

CGM generates the densest physiological time series routinely collected in medicine: one reading every 1–15 min for months per patient, capturing meals, activity, medication, sleep, and illness as a single entangled signal. Era-II CGM modeling was dominated by per-patient or per-dataset supervised models: LSTMs and GRUs trained to forecast glucose 30–60 min ahead, typically on single-study cohorts of tens to hundreds of patients (OhioT1DM, HUPA, Shanghai T1DM cohorts; Marling and Bunescu, 2020; Zhu et al., 2021). The supervised literature exhibits the canonical era-II signature: low intra-dataset RMSE (30–60 min prediction horizons; although RMSE alone is a poor guide to clinical usefulness, see Section 6.1), brittle cross-dataset transfer, and a fragmented ecosystem in which model families rarely re-evaluate on common benchmarks.

The pretraining argument for CGM is therefore unusually clean: the same underlying physiology (glucose–insulin–meal dynamics) generates every stream, while labels (hypoglycemia events, excursions, HbA1c proxies) are scarce and expensive relative to raw signal volume: a near-textbook case for self-supervised representation learning via masked-segment reconstruction or contrastive window prediction.

#### 4.2.2 The current CGM-FM landscape: three technical approaches

The literature has not yet converged on a single CGM-FM recipe; three approaches dominate (Table 5; scale compared in Figure 7):

- Generic time-series FMs applied to CGM. Pretrained temporal transformers (patch-based time-series transformers in the lineage of PatchTST (Nie et al., 2023) and masked-autoencoder time-series models (Zerveas et al., 2021) and general-purpose “time-series FMs” emerging since 2023, e.g., Wu et al., 2023), are adapted to CGM forecasting and hypoglycemia anticipation. Evidence is predominantly L4 (public datasets), with cross-dataset zero-shot forecasting demonstrated for the strongest models: a genuinely new capability relative to era-II per-patient models, but not yet shown to translate into clinical-alert performance (sensitivity/specificity for clinically significant hypoglycemia in ambulatory T2DM).
- Diabetes-native pretrained encoders. Models pretrained exclusively on large CGM corpora (commercial research corpora and aggregated study data; hundreds of millions of patient-hours in aggregate across industry efforts) with objectives combining masked reconstruction and future prediction, adapted to hypoglycemia prediction, glycemic-variability phenotyping, and meal detection. Published peer-reviewed evidence for the greatest industrial efforts is limited; much exists as preprints or non-validated claims, a governance pattern we flag in Section 6.
- Generative CGM models as data infrastructure. Diffusion- and autoregressive-generative models synthesizing realistic CGM streams are increasingly used to augment scarce labeled datasets, evaluate predictors at scale, and share privacy-constrained data (the privacy safeguards are distinguished in Section 7.4). The central open problem is downstream validity: we identified no established standard for certifying that a synthetic CGM corpus preserves the joint distribution of features that downstream clinical models rely on; models trained on synthetic data have shown distributional artifacts in variability statistics and event-timing correlations.

**Figure 7:**
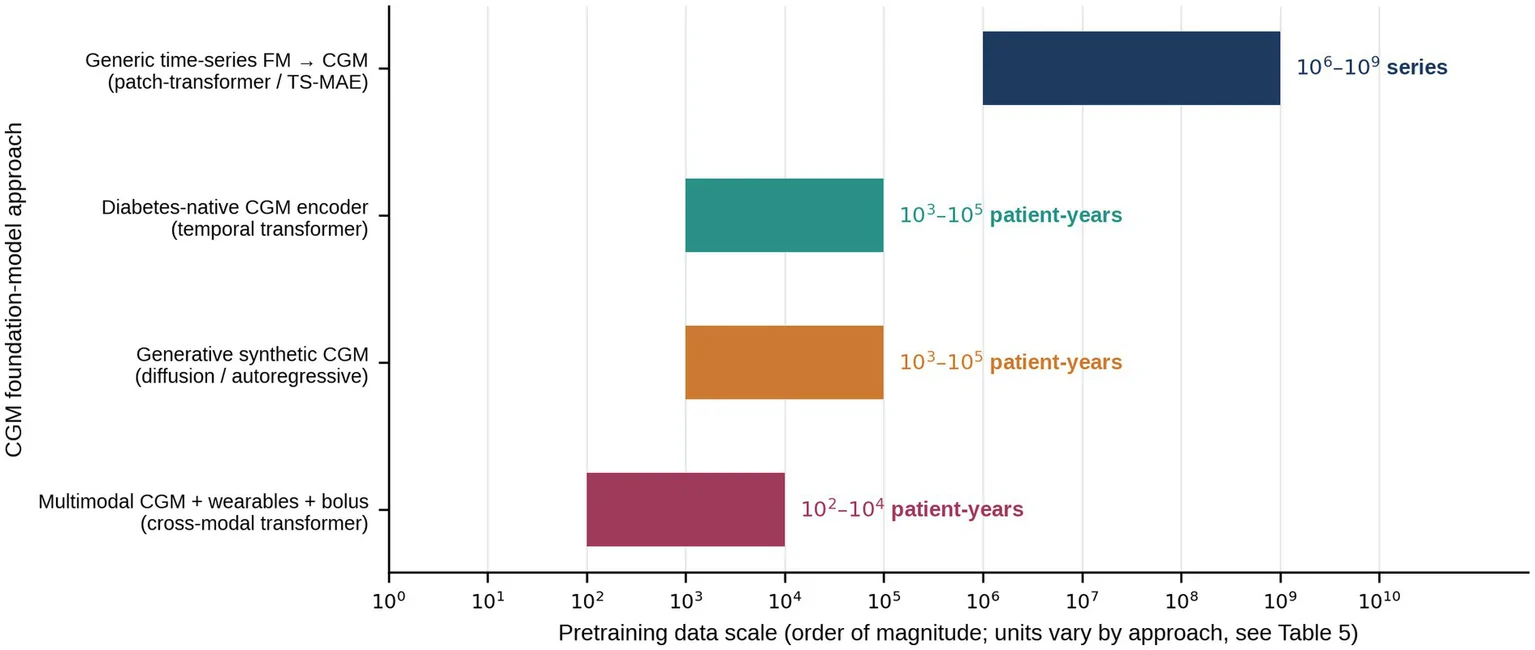
Pretraining data scale by CGM foundation-model approach (Table 5). Order-of-magnitude ranges for generic time-series FMs adapted to CGM, diabetes-native CGM encoders, generative synthetic-CGM models, and multimodal CGM + wearables+bolus models. Units differ across rows (image/series counts versus patient-years), so the chart is a schematic summary, not a harmonized comparison. Source: Authors’ schematic summary of Table 5.

#### 4.2.3 Population and device heterogeneity: T1DM–T2DM, sensor generation, and life stage

A structural blind spot deserves emphasis: the CGM deep-learning literature, and nearly all CGM-FM work to date, is T1DM-centric, reflecting the artificial-pancreas lineage of the field (Man et al., 2014; Kovatchev et al., 2016; Battelino et al., 2019). Yet CGM use is expanding into T2DM and prediabetes, and CGM-FM evaluation has not kept pace. Within the literature we reviewed, we found no CGM-FM evaluation stratified by diabetes type, age band, pregnancy status, or sensor generation. These axes change the signal and the clinical question in different ways, so we take them in turn:

- Sensor generation. Successive CGM generations differ in accuracy (usually summarized as mean absolute relative difference, MARD, and often poorer in the hypoglycemic range that matters most), sampling interval (roughly 1–15 min), sensor lag, calibration requirement (fingerstick-calibrated versus factory-calibrated), warm-up behavior, and data-gap patterns. A corpus that mixes generations mixes measurement distributions, and a hypoglycemia-alert model may learn one device’s error profile rather than physiology. In T1DM, insulin-delivery mode adds a second confound: traces from automated (closed-loop) insulin delivery partly reflect controller behavior and are not interchangeable with those from injection or open-loop users.
- T1DM versus T2DM. Absent endogenous insulin, T1DM produces large excursions, frequent hypoglycemia, and tight dependence on insulin dosing and meals. In T2DM, residual endogenous insulin, oral and incretin pharmacotherapy, slower excursions, less frequent hypoglycemia (concentrated in insulin- and sulfonylurea-treated people), and marked behavioral and phenotypic heterogeneity (Ahlqvist et al., 2018) alter the dynamics. CGM wear also differs: T2DM data are often short, intermittent windows (for example, periodic 10-14-day sensors) rather than continuous months, a mismatch with models pretrained on continuous streams. Whether T1DM-pretrained representations transfer to T2DM regimes is little studied (agenda item A3).
- Children and adolescents. Growth, puberty-related insulin resistance, caregiver-managed meals and dosing, school and sport schedules, and high glycemic variability produce patterns that differ from adults. Consensus time-in-range targets for children and adults are broadly similar (Battelino et al., 2019), but pediatric CGM data are overwhelmingly T1DM, youth-onset T2DM is scarce in the literature, and wear and alert behavior depend on caregivers.
- Pregnancy. Pregnancy, whether with pre-existing T1DM or T2DM or with gestational diabetes, combines tighter targets (for T1DM in pregnancy, a 63–140 mg/dL range with more than 70% time in range; Battelino et al., 2019) with rapidly shifting insulin sensitivity: requirements and hypoglycemia risk change in early pregnancy, insulin resistance rises later, and requirements fall abruptly after delivery. A model trained on non-pregnant adults would apply the wrong thresholds and dynamics, and pregnancy CGM cohorts are small.
- Older adults. Hypoglycemia unawareness, polypharmacy, renal impairment, frailty, and cognitive decline change the risk profile, and consensus targets are relaxed for older or high-risk people (more than 50% time in range, under 1% below 70 mg/dL; Battelino et al., 2019). Older adults are under-represented in CGM research cohorts, so alert thresholds tuned on younger adults may mis-warn them.

These axes interact (for example, a pregnant T1DM user of a recent automated-delivery system), and the smaller strata are often too small for stable estimates. We therefore recommend that CGM-FM reports (i) tabulate pretraining and test data by diabetes type, treatment, age band, pregnancy status, and sensor generation; (ii) report performance and calibration within each stratum, with sample size and uncertainty, rather than only pooled; and (iii) test transfer explicitly (T1DM to T2DM, adult to pediatric, held-out device generation) as part of the standard in Section 6.3. Otherwise, a pooled score can hide failure in the strata where CGM-guided decisions carry the most risk. This is the sharpest single example of the review’s broader claim that era-III capability has outrun era-appropriate evaluation.

### 4.3 Longitudinal EHR: foundation models for structured and narrative patient histories

#### 4.3.1 From feature engineering to sequence modeling

Era-II tabular diabetes modeling used gradient-boosted trees over hand-compiled features (HbA1c trajectory summaries, eGFR slopes, and medication histories). Era-III EHR FMs treat the patient record itself as a language-like or event-like sequence: diagnoses, medications, laboratory results, and notes tokenized and modeled with transformer objectives (next-visit prediction, masked-value imputation, or autoregressive generation). General-purpose clinical FMs in this lineage, trained on millions of patient records, exemplified by Med-BERT (Rasmy et al., 2021) and BEHRT (Li et al., 2020), both adapting the masked-language-modeling objective introduced by BERT (Devlin et al., 2019) to structured clinical sequences and building on foundational clinical-sequence work such as RETAIN (Choi et al., 2016), Doctor AI (Choi et al., 2017), and academic models trained on MIMIC-derived corpora (Johnson et al., 2016), can be adapted to diabetes endpoints: incident DKD prediction, hypoglycemia risk during medication changes, progression of retinopathy stage, and treatment-response trajectory forecasting.

#### 4.3.2 Diabetes-specific technical challenges

Four challenges distinguish EHR-FM adaptation for diabetes from generic clinical-NLP capability:

- Rare-event learning. ESKD and proliferative retinopathy are low-prevalence, long-horizon endpoints; self-supervised pretraining does not automatically confer sample efficiency on events separated from the pretraining signal by years of follow-up. Survival-structured adaptation (time-to-event heads over FM representations) remains largely unexplored relative to classification heads.
- Leakage and coding artifacts. Diabetes endpoints in EHR are heavily mediated by coding behavior (ICD density varies by site and payer); FM representations can latch onto documentation artifacts that vanish across sites: the EHR analog of the camera-shift problem in retinal imaging, and equally unaddressed (L3-level validation dominates).
- Medication-representation grounding. Treatment-response prediction requires representations that distinguish drug exposure from drug indication (confounding by indication is the defining threat to any observational treatment model). Vanilla sequence FMs encode both identically; to our knowledge, no widely adopted diabetes EHR-FM yet incorporates treatment-aware or causal pretraining objectives: the technical seam where Sections 4.3 and 8 (research agenda, item A10) meet.
- Text + structured fusion. The highest-signal diabetes content (podiatric exam notes, ophthalmology letters, hypoglycemia narratives) lives in text; the highest-reliability longitudinal signal lives in structured data. Multimodal EHR FMs addressing both exist in early form (Shickel et al., 2018); diabetes-specific validation is L4 at best.

### 4.4 Cross-modality synthesis and the multimodal frontier

Across all three modalities the same three-part pattern recurs: a pattern that constitutes the review’s central empirical observation about era III:

- Architecture convergence, corpus divergence. Every modality has converged on transformer backbones (Vaswani et al., 2017); all domain specificity now resides in the pretraining corpus and objective. Consequently, corpus curation (not architecture) is the active scientific variable in diabetes FMs, with direct consequences for bias, coverage, and reproducibility (Sections 6–7).
- The label-efficiency dividend is consistently reported, chiefly by model developers. Across retina (RETFound), CGM, and EHR, developers report that pretrained backbones match fully supervised performance with substantially fewer task labels, in some reports by one to two orders of magnitude. Independent retinal comparisons find the advantage confined largely to label-scarce settings (Section 4.1.3), and we identified no comparable independent comparisons against tuned classical baselines for CGM or EHR. This is the most consistently reported technical theme of the era-III diabetes literature and should be read with Section 4.5.
- Validation level has not advanced with capability. In Tables 4–6, the evidence is mostly L3–L4 (a few retinal tasks reach L2), and L1 is, to our knowledge, absent (Section 6.2).

The multimodal frontier: joint pretraining or alignment across retina, CGM, EHR, and text toward a unified patient representation is the logical terminus of this trajectory and the least mature: to our knowledge, no multimodal diabetes FM in the literature reviewed has external (L2) validation on any diabetes endpoint; the evidence is limited to internal (L3) and benchmark-only (L4) evaluation. The integration points, however, are already clinically meaningful: retinal-FM embeddings correlated with CGM-derived variability phenotypes, or CGM-FM representations conditioning EHR-FM risk forecasts, would operationalize the multisystem view of diabetes that era-II single-task models could not express. We formalize this as agenda item A4 (Section 8); the label-efficiency dividend underlying this trajectory is summarized in Figure 8.

**Figure 8:**
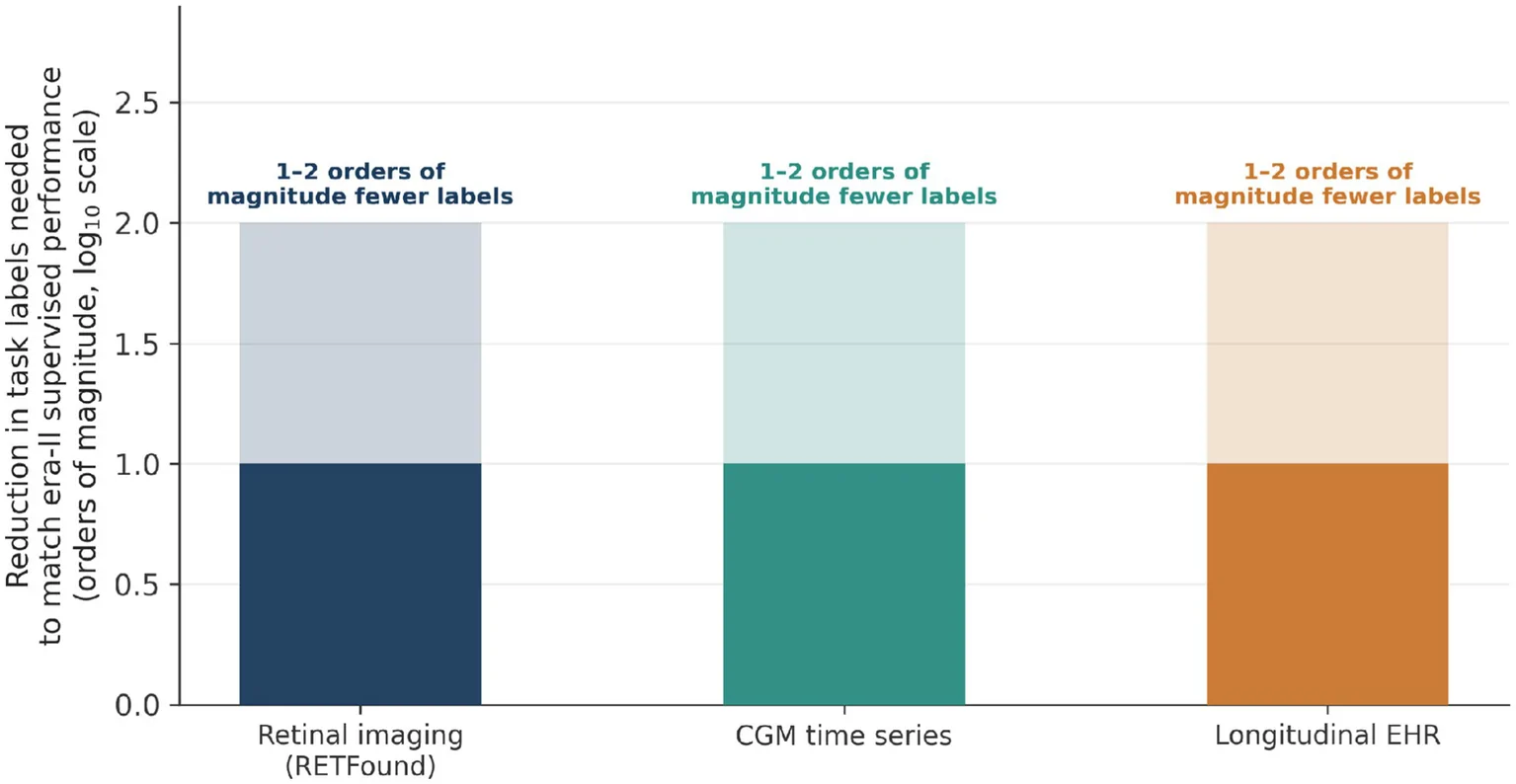
Label-efficiency dividend across diabetes foundation-model modalities. Across retinal imaging (RETFound), CGM time series, and longitudinal EHR, pretrained backbones are reported to match fully supervised era-II performance with roughly one to two orders of magnitude fewer task labels (Section 4.4); the claim is made largely by model developers and is best supported when labels are scarce. Source: Authors’ schematic summary of ranges reported in Sections 4.1–4.3; not a pooled estimate.

### 4.5 Interpretability, explainability, and inductive bias

#### 4.5.1 Interpretability and explainability

Era-I risk scores are interpretable by construction. Era-II models were typically explained *post hoc* (saliency or Grad-CAM maps for retinal graders, feature attributions for tabular risk models). Era-III models add new problems: attention weights do not reliably explain a decision (Jain and Wallace, 2019), and the rationales produced by LLMs can be fluent yet unfaithful to the computation that produced the answer (Turpin et al., 2023). *Post hoc* explanation methods have also been criticized as unreliable for high-stakes clinical use, with researchers proposing inherently interpretable models where possible (Rudin, 2019; Ghassemi et al., 2021). For clinically applied diabetes models, this matters most where a clinician must decide whether to trust an output, for example, a referral decision from a retinal FM or an insulin-related suggestion from an LLM. Explanation quality was not part of the analytic frame of this review (Section 2.4); we flag the lack of faithfulness testing and clinician-facing evaluation of explanations as a gap, and we recommend that explanation reporting be added to the evaluation standard of Section 6.3.

#### 4.5.2 Inductive bias and accessibility

Convolutional networks build in locality and translation equivariance, and recurrent networks build in sequential order. Transformers impose far fewer such image- or sequence-specific assumptions and rely on very large pretraining corpora and compute to learn structure from data (Dosovitskiy et al., 2021). This is the technical core of the “architecture convergence” noted in Section 4.4, and it has a practical consequence: pretraining at scale is out of reach for most research groups, and in the small cohorts typical of diabetes (single-study CGM datasets, single-center imaging sets), well-tuned classical networks and physiology-informed hybrid models can remain competitive; in retinal imaging, ImageNet-pretrained supervised networks were largely comparable to RETFound for ocular disease detection when labeled data were plentiful (Yew et al., 2025). Claims of FM superiority should therefore be tested against strong, tuned era-II and interpretable baselines trained on the same data and compute budget, and the label-efficiency dividend (Figure 8) should be read with that comparison in mind.

## 5 Clinical large language models in diabetes care

### 5.1 Capability: what LLMs demonstrably know about diabetes

Clinical LLMs have crossed medical licensing-exam performance thresholds on benchmarks containing substantial diabetes-management content, part of a broader capability trajectory for LLMs in medicine (Thirunavukarasu et al., 2023). Med-PaLM reached 67.6% on MedQA (USMLE-style), and Med-PaLM 2 reached 86.5% (Singhal et al., 2023a, b); GPT-4-family models reported comparable or higher performance on MedQA and related suites (Nori et al., 2023). Long-form answer quality has been evaluated against clinician reference panels: on the MultiMedQA evaluation, physician graders rated Med-PaLM answers favorably across axes, including comprehension of diabetes-management questions, though with systematic gaps in currency of guideline detail and in harm-avoidance framing (Singhal et al., 2023a). In adjacent patient-facing settings, blinded comparisons have rated chatbot responses to health questions as more empathetic than physician responses in some contexts (Ayers et al., 2023), underscoring that fluency and bedside manner are not proxies for clinical accuracy.

Three technical points qualify every such score:

- Benchmark structure rewards retrieval-shaped knowledge. USMLE-style items test guideline-recall reasoning over well-posed vignettes. Diabetes management in the wild is the opposite: a long-horizon, multi-signal, titration problem (which drug sequence, at what HbA1c trajectory, with which comorbidity constraints) that, to our knowledge, no benchmark yet operationalizes.
- Contamination is plausible and largely unmeasurable for closed models. Licensing-exam content and its explanations circulate massively on the web: the same corpora on which these models are pretrained (Section 6.2). Exam scores are therefore an upper bound on, not an estimator of, operational competence.
- Versions are non-stationary. Closed-weight models are updated without versioned clinical re-evaluation; a diabetes-care benchmark score from one model generation does not bind its successor (Figure 9 plots the MedQA progression discussed above).

**Figure 9:**
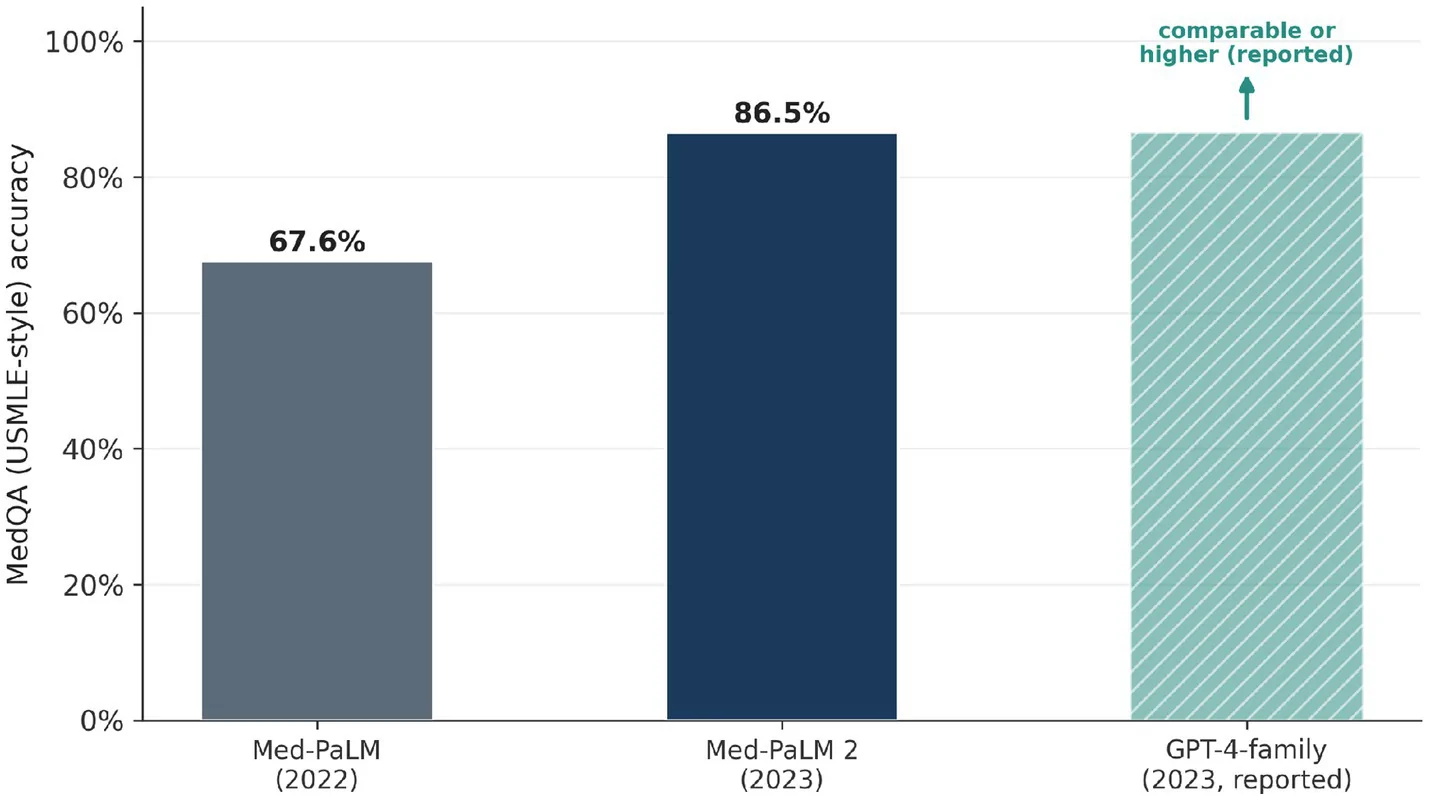
MedQA (USMLE-style) performance of clinical LLMs. Med-PaLM (67.6%, 2022) to Med-PaLM 2 (86.5%, 2023), with GPT-4-family models reported as comparable or higher. Exam accuracy is an upper bound on, not an estimator of, operational diabetes-management competence (Section 5.1). Source: Singhal et al. (2023a, b) and Nori et al. (2023), redrawn by the authors.

### 5.2 The adaptation stack: how LLMs are being specialized for diabetes

The engineering surface for LLM deployment in diabetes care has stratified into a recognizable stack, each layer trading capability for verifiability (Table 7):

- Prompt-level adaptation (zero/few-shot, chain-of-thought). Cheapest, no training, but output stability is prompt-sensitive, and no institution controls behavior.
- Retrieval-augmented generation (RAG) (Lewis et al., 2020). Grounds generation in a controlled corpus: the ADA Standards of Care (American Diabetes Association Professional Practice Committee for Diabetes, 2026), local formularies, and patient record context. RAG directly attacks the currency problem (guidelines update annually; weights do not) and mitigates some hallucination by constraining generation to retrieved evidence but introduces retrieval-failure modes: wrong-chunk selection, stale indexed guidelines, and confident synthesis across contradictory retrieved passages.
- Parameter-efficient fine-tuning (low-rank adaptation, LoRA, and other adapter methods; Hu et al., 2022) on diabetes-specific corpora, following the pattern of open, fully fine-tuned medical LLMs such as Clinical Camel (Toma et al., 2023). Clinical dialogue datasets, endocrinology consult notes, and diabetes education transcripts. Improves domain register but risks amplifying corpus biases and is institution-specific and unsuitable for regulators at scale.
- Agentic orchestration. LLM planners invoking tools: CGM-data interpreters, drug-interaction checkers, EHR query interfaces, and insulin-dose calculators (deterministic tools). The agent paradigm is where the field is heading for safety reasons: it converts free-text generation into constrained tool calls, allowing deterministic components to enforce hard safety constraints (e.g., maximum dose bounds) that the LLM itself cannot guarantee.

### 5.3 The deployment surface: where LLMs actually touch diabetes patients

We identify four deployment classes in ascending order of risk; the classes and their risk ordering are the authors’ proposal, not a clinically validated taxonomy (Section 2.1):

- D1: Clinician-facing documentation and information retrieval. Ambient documentation, note summarization, and guideline lookup. Risk profile comparable to other clinical software; already commercially deployed at scale.
- D2: Clinician-facing decision support. Drafting differential considerations, flagging guideline-discordant medication choices. Intermediate risk: automation bias (Section 7.2) applies.
- D3: Patient-facing education and navigation. Diet, medication-adherence messaging, and appointment triage. Already deployed by commercial diabetes platforms; safety evidence is observational at best.
- D4: Patient-facing autonomous advice on glycemic management. Any system that, without clinician mediation, advises on insulin dosing, sulfonylurea changes, or hypoglycemia response. We identified no L1 evidence for any system in this class, and Section 7.1 documents why the failure modes here are not merely theoretical.

The asymmetry across D1–D4 is the section’s conclusion: LLM capability is roughly uniform across classes; LLM safety evidence is concentrated entirely in D1. Commercial pressure pushes deployment toward D3–D4. This divergence (capability uniform, evidence gradient) is a governance gap specific to the current moment.

## 6 The evaluation crisis: how era-III evidence is broken

### 6.1 Five failure mechanisms

- Benchmark contamination: For LLMs, pretraining corpora scrape the same public web on which benchmark items (MedQA and derivatives, diabetes exam question banks) circulate. Closed-weight models do not disclose corpora, so contamination cannot be excluded; where audits have been attempted (canary-string insertion, perturbation-robustness testing with paraphrased, order-shuffled, or numerically altered variants), performance drops materially, consistent with memorization contributing to headline scores (cf. Ji et al., 2023 on hallucination and related failure modes).
- Cohort entanglement for imaging/temporal FMs: RETFound’s pretraining (UK Biobank, Moorfields) overlaps its evaluation populations; the same holds for nearly every diabetes CGM and EHR FM. “External validation” in much of this literature means external site only (a different institution or device), not external population or later era (Section 2.3).
- Metric misalignment: AUROC and RMSE dominate reporting; the metrics that determine clinical usability (sensitivity at program-specified specificity, calibration curves, decision-curve analysis, net benefit at screening-program thresholds) are reported in a small minority of era-III studies, and a perfectly discriminative but miscalibrated FM is dangerous at a referral threshold. For CGM forecasting, RMSE and MAE summarize point error but not clinical risk and should not stand alone; clinically anchored evaluation should accompany them: Clarke or Parkes error-grid analysis (Clarke et al., 1987; Parkes et al., 2000), time-in-range–based outcomes (Battelino et al., 2019), and hypoglycemia-alert sensitivity, precision, and lead time.
- Version non-stationarity: Closed-model benchmark results do not transfer across model updates; there is, to our knowledge, no settled re-certification norm (although FDA change-control-plan guidance now offers a pathway; Section 7.5), in contrast to drug manufacturing, where change control is mandatory.
- Absence of outcome-level evaluation: To our knowledge, no published randomized trial had evaluated an FM as the interventional component of a diabetes workflow. A ClinicalTrials.gov search in August, 2026 identified one relevant registration, a cluster-randomized trial of an LLM-enabled coordinated-management tool for hypertension, diabetes, and dyslipidemia (NCT07350486), not yet recruiting at the time of the last check; no completed or reported outcome-level evaluation of an FM in a diabetes workflow existed as of that search. Given the pace of trial registration, this time-sensitive finding should be re-verified close to publication.

### 6.2 Evidence levels across the reviewed literature

Applying the L1–L4 grading of Section 2.3 to the studies examined in Sections 4–5 (Tables 4–7) gives a consistent qualitative pattern, which we present as a critical appraisal rather than a quantified audit. In the literature we reviewed, we found no L1 studies; external validation (L2) was uncommon; most diabetes foundation-model studies were validated at L3 (internal held-out data) or L4 (benchmark-only); and calibration reporting and fairness subgroup analysis were rare.

#### 6.2.1 Reading this appraisal

No counts or percentages are claimed, and the appraisal is bounded by Section 2.1 and time-sensitive. Individual models do reach L2 (RETFound is graded L2 on three retinal tasks in Table 4), but, as Section 2.3 explains, L2 may rest on external-site evidence alone.

Table 3 summarizes descriptively the principal studies on which Sections 4–6 rely. It is not a complete inventory of the literature reviewed and not a quality ranking; levels are the authors’ interpretive grading from what each report states (Section 2.3), and the final column records the limitations discussed in the text.

Three conclusions follow. First, the capability literature and the validation literature have decoupled: the strongest models in Tables 4–6 are mostly validated at L3–L4 (a few retinal tasks reach L2), while L1 is, to our knowledge, empty across the reviewed landscape. Second, the era-II field’s hard-won lesson [that Gulshan-style internal AUROCs fall under site shift (Finlayson et al., 2021)] has been forgotten rather than institutionalized: era-III papers inherit the era-II evaluation style without era-II correction. Third, the retinopathy screening program is, to our knowledge, the only domain where the full evidence ladder (L1–L2 deployment at scale; IDx-DR’s pivotal trial, Abràmoff et al., 2018) was ever climbed, and that climb happened under era-II, task-specific conditions. We found nothing in era III that has repeated it. The resulting capability–validation divergence is illustrated in Figure 10.

**Figure 10:**
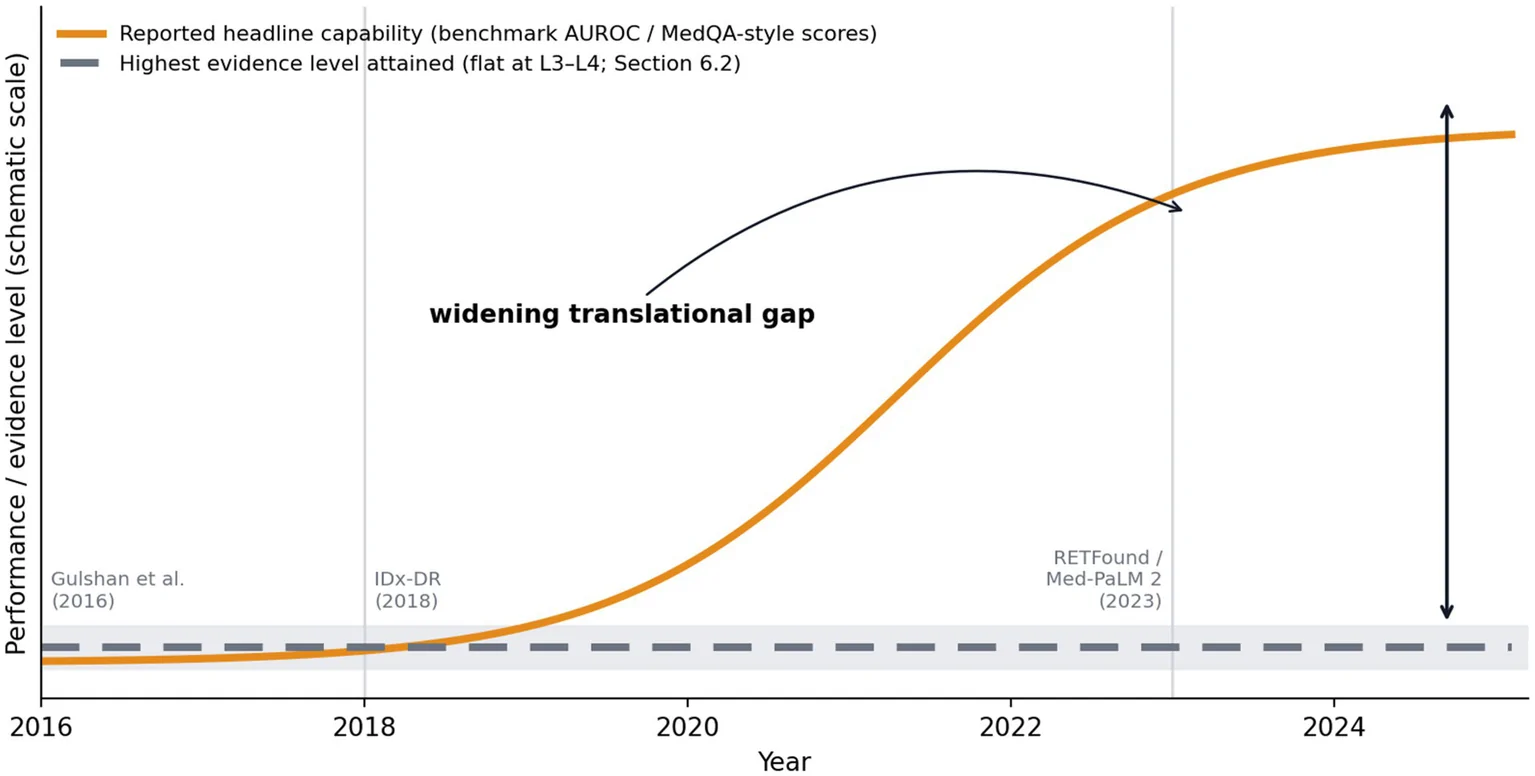
Capability–validation divergence, 2016–2025 (schematic). Reported headline capability rises while the highest evidence level attained stays flat at L3–L4, illustrating the widening translational gap (Section 6.2). Source: Authors’ conceptual illustration of the qualitative pattern in Section 6.2; curves are not derived from empirical data or a quantitative meta-analysis.

### 6.3 What usable evaluation would require

We specify, concretely, the evaluation standard that the era-III diabetes literature currently lacks, anchored to the TRIPOD+AI reporting standard (Collins et al., 2024; Collins et al., 2015):

- Pre-registered, frozen test sets with temporal separation (train-before/test-after) and population provenance disclosure for every modality.
- Contamination disclosure for LLM evaluations: corpus-to-benchmark overlap statements, or perturbation-robustness results, as a minimum proxy for closed models.
- Mandatory calibration reporting: calibration curves, expected calibration error, and decision-curve analysis at the clinically operative threshold (screening sensitivity/specificity targets; hypo-alert thresholds).
- Subgroup analysis as default: performance disaggregated by age, sex, ethnicity, diabetes type and treatment, pregnancy status, device generation (CGM; Section 4.2.3), camera field (retina), and site.
- Version-pinned results: all closed-model benchmark reports must state the exact model version and date; authors of downstream deployments must re-evaluate on update.
- Prospective silent-trial mode as the default intermediate step before L1: the model runs in shadow mode alongside clinicians, with concordance and override-rate analysis reported.
- External validation reported by type: external-site, external-population and temporal validation reported separately, because passing one does not show transport across the others (Section 2.3).
- Explanation reporting: where a model output is presented with an explanation (saliency map, attention weights, or LLM rationale), report faithfulness testing and clinician-facing evaluation of whether the explanation improves or degrades decisions (Section 4.5).
- Tuned baselines: foundation-model claims compared against strong, tuned era-II and interpretable baselines trained on the same data and compute budget, with performance reported across label fractions (Section 4.5).

None of these nine items is technically difficult; their absence is cultural, not computational (pipeline in Figure 11).

**Figure 11:**
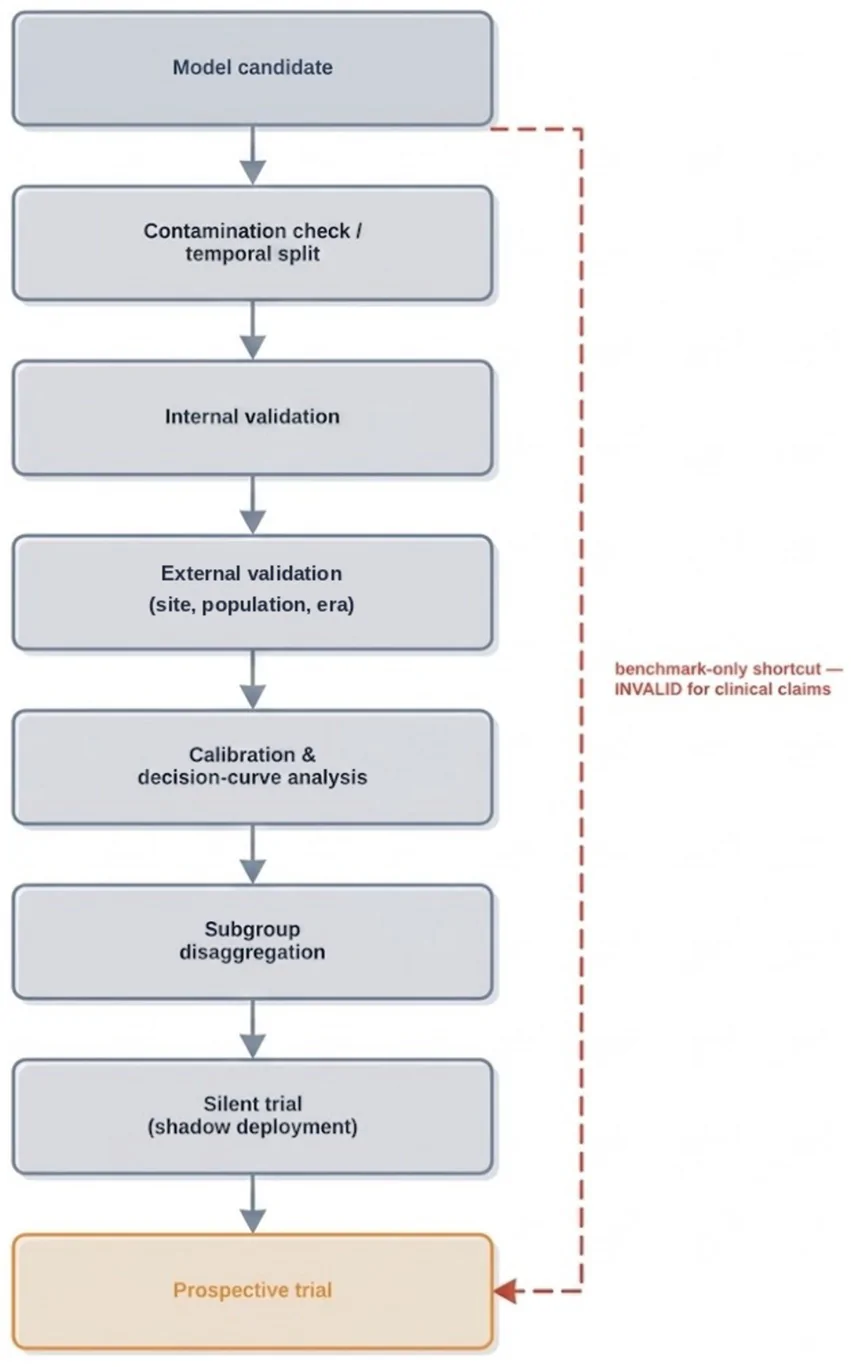
Proposed evaluation pipeline (Section 6.3 standard). Model candidate → contamination check/temporal split → internal validation → external validation (site, population, era; Section 2.3) → calibration and decision-curve analysis → subgroup disaggregation → silent trial → prospective trial; the benchmark-only shortcut is marked invalid for clinical claims. Source: Authors’ proposed pipeline; conceptual diagram.

## 7 The safety frontier: hallucination, automation bias, inequity, privacy

### 7.1 Hallucination in the glycemic-critical zone

Hallucination [fluent, confident generation unsupported by input or fact (Ji et al., 2023)] is a general LLM pathology with diabetes-specific lethality gradients. The severity gradient tracks Section 5.3’s D1–D4 classes, with risk concentrating in D3–D4 as summarized in the safety matrix (Figure 2; Table 2):

- In D1–D2 (clinician-facing), hallucinated content is largely intercepted by professional review; the residual risk is automation bias (Section 7.2).
- In D3–D4 (patient-facing), interception is, to our knowledge, absent in the systems we reviewed. The critical zone is glycemic-critical content: insulin doses, hypoglycemia response, sick-day rules, and medication interactions. Rigorous, systematically documented evaluations of LLM performance specifically on diabetes medication-management questions remain sparse (itself symptomatic of the evaluation crisis this review documents in Section 6), but the failure pattern one would predict from general-purpose LLM hallucination behavior is errors in dosing advice and outdated or contraindicated recommendations under specific prompts. The structural problem is that hallucination probability is not uniformly distributed: it concentrates precisely where training data are sparse, conflicting, or rapidly changing: insulin titration under renally declining patients, perioperative management, and steroid-induced hyperglycemia, i.e., exactly the long-tail cases where clinical judgment matters most.

Two technical mitigations have partial evidence: retrieval grounding (Section 5.2) reduces (but does not eliminate) factual hallucination; and constrained decoding over structured dose spaces (tool-enforced bounds) can make certain hallucination classes impossible. Neither approach has been evaluated in any prospective diabetes workflow. The research gap is precise and CS-native: hallucination quantification conditioned on content criticality (measuring not overall hallucination rate but rate restricted to glycemic-critical spans), and we formalize it as agenda item A6.

### 7.2 Automation bias and the erosion of clinical vigilance

Automation bias (the tendency of human operators to over-trust automated outputs) is documented in era-II clinical AI deployments (radiology triage, sepsis alerts; Beede et al., 2020) and applies with additional force to era III because LLM outputs are linguistically fluent, and fluency is a known cue for perceived competence. In diabetes care, the highest-risk interaction is guideline-discordant medication selection proposed confidently in D2 workflows; a clinician under time pressure must actively override, and override behavior is known to decay with alert volume. We identified no study that has measured automation bias for foundation-model decision support in endocrinology. The measurement instrument (override-rate telemetry, calibrated-confidence display, forced-friction designs) exists in the human-factors literature (Sutton et al., 2020); its application here is agenda item A7.

### 7.3 Algorithmic inequity at foundation-model scale

Era II diabetes AI inherited dataset bias (fundus datasets skewed to screened populations; CGM cohorts skewed to insured T1DM populations); era III industrializes the inheritance. Two mechanisms are new in kind:

- Corpus-level bias at the pretraining scale. Skews in pretraining corpora (which populations get imaged, monitored, and documented) are absorbed into representations and propagate silently through every downstream adaptation. Unlike a fine-tuned era-II model (whose training set can be audited), a foundation model’s corpus is typically undisclosed (closed models) or too broad to control (open models), making bias auditing *post hoc* and statistical rather than mechanistic (Mehrabi et al., 2021).
- Benchmark-blindness to inequity. The benchmarks driving era-III progress (MedQA, EyePACS leaderboard metrics) carry minimal subgroup stratification; a model can top every headline leaderboard while being materially worse for the populations carrying the highest diabetes burden (Section 6.3). Performance-under-subgroup-shift studies in era-II DR screening documented material AUROC degradation across demographic and device shifts (e.g., Beede et al., 2020); we identified no era-III diabetes FM report with equivalent disaggregation at scale.

The equity consequence compounds the access analysis familiar from health-services research (Obermeyer et al., 2019; Rajkomar et al., 2018): if FMs are deployed first in well-resourced, well-documented health systems, pretraining–deployment loops will reinforce the very data asymmetries that made representations weak for high-burden populations in the first place: a feedback ratchet that era II, lacking pretraining loops, could not exhibit (Figure 12 diagrams this ratchet).

**Figure 12:**
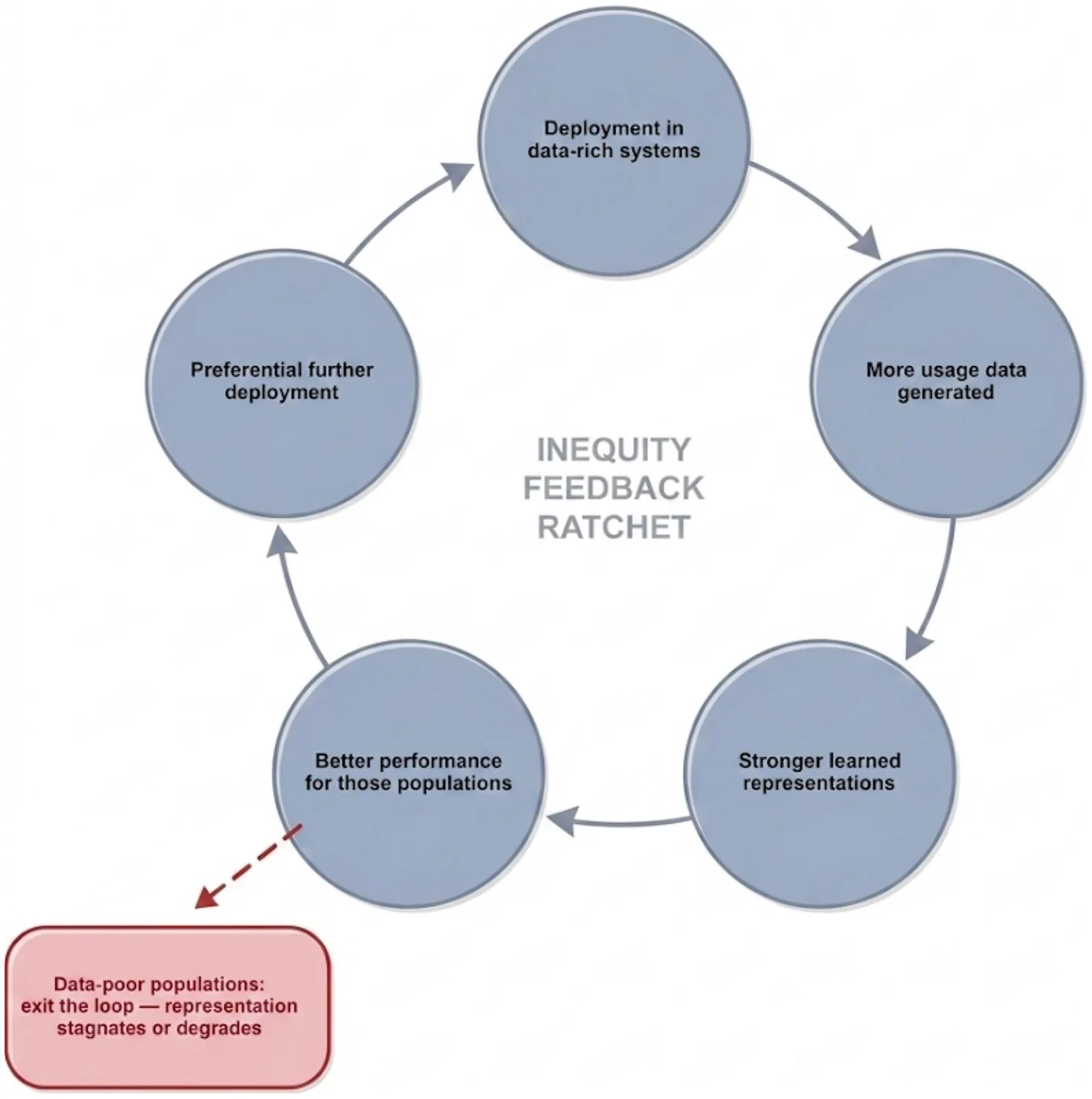
Inequity feedback-ratchet loop. Deployment in data-rich systems generates more data, stronger representations, and better performance for those populations, while data-poor populations exit the loop (broken arrow). Source: Authors’ conceptual diagram (Section 7.3); no data.

### 7.4 Privacy: the new exposure surface

Foundation models introduce privacy exposures that era-II supervised learning did not:

- Adaptation leakage: Fine-tuning a general FM on institutional records can leak memorized patient content through prompted extraction, as documented for large LLMs generally (Carlini et al., 2021).
- Inference-time leakage: Patient data submitted to closed-weight, API-hosted models leaves the institutional boundary; contractual de-identification is the current norm, but re-identification risk for longitudinal diabetes records (dense, quasi-identifying: CGM timing signatures, rare medication combinations) is understudied.
- Synthetic data’s unproven guarantee: Generative synthetic CGM/EHR (Sections 4.2–4.3) is increasingly marketed as privacy-preserving, but such claims rest on three different safeguards that are often conflated, and most published generative-diabetes pipelines report none of them with parameters (cf. Abadi et al., 2016; Walonoski et al., 2018). They answer different questions and are not interchangeable:
- De-identification is a data-handling step: it removes or transforms direct identifiers (names, record numbers, and exact dates) in source or released records. It carries no formal guarantee. Dense longitudinal diabetes data (CGM timing signatures, rare medication combinations) can re-identify people through quasi-identifiers, and a generative model trained on de-identified records can still reproduce them.
- Differential privacy (DP) is a property of the training or generation algorithm. It bounds, through a parameter ε (with δ), how much any one individual’s data can change the output (Abadi et al., 2016). It is a formal guarantee that does not depend on the attacker, usually at some cost in utility, and it is only interpretable when the privacy unit (a record, a visit, or a whole patient; the last matters for longitudinal data) and the ε value are stated.
- Memorization audits are empirical tests of one trained model: whether it leaks training records, for example, through training-data extraction, membership inference or nearest-neighbor distance-to-training-record tests (cf. Carlini et al., 2021). They can reveal leakage but cannot prove its absence, because a negative result holds only for the attacks tried.

A pipeline can have any one of these without the others: a DP-trained generator may be unaudited, and an audited generator has no formal bound. Synthetic-data claims should therefore state which safeguard applies and with what parameters (ε, δ, and privacy unit; the attacks run and their results; the de-identification standard used). Rule-based generators such as Synthea (Walonoski et al., 2018) avoid training on real records by construction, which provides a different privacy basis than DP.

Federated adaptation (fine-tuning across institutions without centralizing data, with secure aggregation and differential privacy (DP) noise) is the technically coherent response and exists in early clinical-FL deployments (McMahan et al., 2017; Rieke et al., 2020; Dayan et al., 2021), but the intersection of federated × foundation-model × diabetes remains largely unpopulated (agenda item A8).

### 7.5 Regulatory, governance, and implementation context

#### 7.5.1 Regulation

In the United States, FDA oversight combines clinical decision support guidance, device pathways such as the IDx-DR *De Novo* authorization discussed above, and, for models that are updated, Predetermined Change Control Plans (US Food and Drug Administration, 2024): a plan agreed before marketing that specifies the anticipated modifications, the protocol for developing, validating, and implementing them, and an assessment of their impact. This directly relates to the version-pinning requirement of Section 6.3, since an advance change plan is the regulatory counterpart of re-evaluating on every model update, but it presupposes a documented training and update process that closed, API-hosted FMs often do not expose. In the European Union, AI that is itself a regulated medical device, or a safety component of one, is classed as high-risk under the AI Act (European Parliament and Council of the European Union, 2024a), in parallel with the Medical Devices and *In Vitro* Diagnostic Medical Devices Regulations; the high-risk requirements include risk management, data governance, technical documentation, transparency, human oversight, accuracy and robustness, and post-market monitoring. Providers of general-purpose AI models, the category to which the FMs discussed here belong, are subject to separate obligations that have applied since August 2025. The application date for high-risk AI embedded in regulated products such as medical devices has shifted: it was originally 2 August 2027 and was provisionally deferred to 2 August 2028 in an agreement between the Council and the Parliament in May 2026 (Council of the European Union, 2026). At the international level, the International Medical Device Regulators Forum terminology and principles for machine-learning-enabled medical devices (International Medical Device Regulators Forum, 2022) and the WHO guidance on the ethics and governance of AI for health, including large multimodal models (World Health Organization, 2024), provide shared vocabulary and principles but are not binding.

#### 7.5.2 Post-market surveillance and drift monitoring

Premarket evidence does not settle behavior after deployment. FM-based tools need post-market surveillance with predefined drift triggers: track performance and calibration across CGM device generations, camera types, sites, and subpopulations; changes of base model or prompt version; and adverse events and near misses captured. The AI Act and the medical-device regimes each require post-market monitoring in some form, but, to our knowledge, neither specifies drift metrics suited to a model whose outputs are free text. We suggest, as a minimum, a locally held reference set re-scored on a schedule, subgroup-stratified calibration tracking, and a defined route from a drift signal to the change plan described above.

#### 7.5.3 Liability and accountability

Accountability is currently unsettled for closed, API-hosted models, where the developer, the deploying institution, and the clinician each control different parts of the system. In the European Union, the revised Product Liability Directive (European Parliament and Council of the European Union, 2024b) extends no-fault producer liability to software, including AI systems, so the allocation of responsibility between model provider and deploying institution becomes a question of contracts and evidence (model versions, logs, documented oversight) as much as of clinical judgment. We therefore recommend that deployments name the accountable clinician and institution, retain an audit log of model version and output for each decision, and allocate responsibility for model updates contractually. Liability rules differ by jurisdiction and are changing, and this review does not provide legal advice.

#### 7.5.4 Implementation, workflow, and cost-effectiveness

Implementation science is largely absent from the diabetes FM literature we reviewed. Whether a tool is used safely depends on workflow integration (where in the visit or alert pathway the output appears, who acts on it, and what happens when it is wrong), alert burden (hypoglycemia alerts and patient-facing LLM output are both prone to alert fatigue and to the automation bias of Section 7.2), clinician training, and cost. The economic evidence that exists comes mainly from AI-assisted retinal screening: a modeling study of the Singapore national program found a semi-automated deep-learning model to be the least costly of the screening models compared (Xie et al., 2020), but that is a task-specific model, not evidence for a general-purpose FM. Frameworks such as NASSS, which explains non-adoption, abandonment, and failure to scale (Greenhalgh et al., 2017), and the DECIDE-AI reporting guideline for early-stage live evaluation of AI decision support (Vasey et al., 2022), offer structures for evaluating FM tools in real workflows before and alongside trials.

#### 7.5.5 Equity and low- and middle-income settings

Equity assessment should use an explicit framework such as PROGRESS-Plus (O’Neill et al., 2014; place of residence, race and ethnicity, occupation, gender, religion, education, socioeconomic status, social capital, plus age and disability). Deployment in low- and middle-income settings, where diabetes burden is growing and CGM and retinal-imaging access is limited, needs separate evaluation rather than extrapolation from high-income cohorts. It raises constraints that high-income validation does not test: intermittent connectivity and limited compute, which make cloud-hosted frontier models impractical in some settings; languages poorly represented in pretraining corpora; and limited referral and treatment capacity downstream of an alert. A human-centered evaluation of a retinal deep-learning system deployed in Thai clinics illustrates how image quality, connectivity, and workflow shaped real-world performance (Beede et al., 2020).

## 8 A research agenda for foundation models in diabetes care

The agenda below is organized around the review’s central claim: representation learning has been solved well enough; evaluation, grounding, and governance have not. Items are ordered by dependency; earlier items are prerequisites for later ones, and each is stated as a concrete, executable research program for computer science groups, with the corresponding clinical stake made explicit.

- A1: Contamination-proof diabetes evaluation suites: Construct and maintain living diabetes benchmarks for all three modalities: (i) LLM suites with paraphrase-perturbation, numeric-perturbation, and canary-based contamination tests, refreshed on an annual guideline cycle (the ADA Standards of Care update is a natural versioning clock); (ii) retinal and CGM test sets with temporal separation (data acquired after model release) and disclosed population provenance. Deliverable: a publicly versioned benchmark with contamination disclosures attached to every leaderboard entry.
- A2: Calibration and decision-theoretic evaluation as the default: Replace headline AUROC with threshold-anchored reporting: sensitivity at program specificity, calibration curves and ECE across sites, and decision-curve analysis/net benefit at the operative referral threshold. Methodologically trivial; culturally uphill; publishable as an empirical audit.
- A3: T2DM-native CGM foundation models: Pretrain and evaluate CGM representations on T2DM and prediabetes regimes (heterogeneous oral/incretin pharmacotherapy, slower dynamics, older populations), with stratified pediatric, pregnancy, and device-generation evaluation (Section 4.2.3), and formally test T1DM → T2DM transfer of existing representations. This is the sharpest identified gap in the entire landscape (Section 4.2.3).
- A4: Multimodal diabetes patient representations: Align retinal-FM, CGM-FM, and EHR-FM embeddings toward a unified patient representation, with the flagship diabetes task being DKD trajectory prediction from retina + EHR + CGM jointly, including the retinal→renal transfer task identified as absent in Section 4.1.4. We begin with L2–L3 honestly labeled; the contribution is the first externally validated multimodal diabetes FM, which, to our knowledge, does not currently exist at any level in the literature we reviewed (the proposed architecture is shown in Figure 13).
- A5: Survival-structured and time-to-event adaptation of EHR FMs: Move diabetes EHR adaptation from classification heads to time-to-event heads (incident DKD, retinopathy progression, first ulceration), handling censoring, competing risks (non-diabetes mortality), and the rare-event regime.
- A6: Criticality-conditioned hallucination measurement: Define a hallucination taxonomy over diabetes-advice content stratified by clinical criticality (glycemic-critical spans vs. informational spans) and measure hallucination rate within the critical stratum for D3/D4-class systems, across prompting, RAG, and tool-constrained variants.
- A7: Automation-bias measurement in endocrinology: Adapt human-factors instrumentation (override telemetry, confidence display variants, forced-friction designs) to D2-class FM decision support in diabetes medication management; the first prospective human-factors study of FM assistance in endocrinology would be a landmark regardless of result direction.
- A8: Federated foundation-model adaptation for diabetes: The intersection of federated learning, differential privacy, and FM fine-tuning on diabetes data (Section 7.4) is, to our knowledge, sparsely populated in the literature we reviewed. Concrete first target: federated LoRA adaptation of a shared EHR FM across multi-site diabetes cohorts for incident-DKD prediction, with DP guarantees and memorization audits.
- A9: Prospective silent trials, then the first FM RCT in diabetes: Step 1: shadow-mode deployment of a diabetes FM (e.g., RAG-grounded guideline-concordance support in D2) with concordance/override telemetry across ≥2 sites. Step 2: a cluster-randomized trial with clinician time-to-decision or guideline-concordance rate as an endpoint, with patient outcomes as secondary.
- A10: The causal layer: Couple FM representations with treatment-effect estimation (heterogeneous-effect models over FM embeddings; off-policy evaluation for treatment sequencing). The clinically decisive question in diabetes (which drug sequence for which phenotype) is a treatment-effect question, and, to our knowledge, no current FM architecture answers it. A10 is the highest-risk, highest-reward item and the one most likely to define the field’s next era. Figure 14 maps the dependency relationships among agenda items A1–A10.

**Figure 13:**
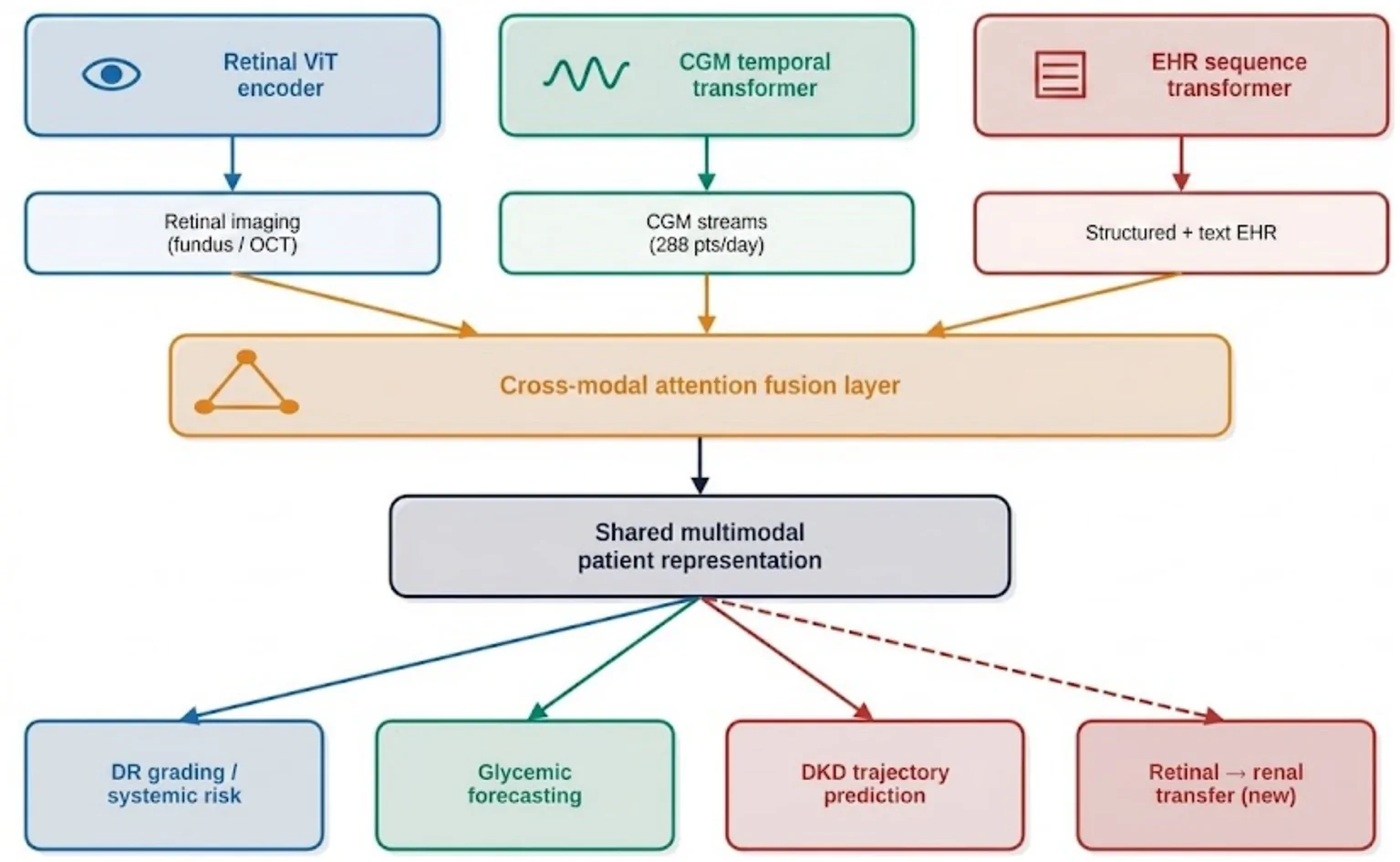
Proposed multimodal diabetes foundation-model architecture (agenda item A4). Three encoder towers (retinal ViT, CGM temporal transformer, and EHR sequence transformer) feed a cross-modal attention fusion layer, a shared patient representation, and task heads; the retinal-to-renal head is marked as not yet existing in the literature. Source: Authors’ proposed design; not an implemented or evaluated model.

**Figure 14:**
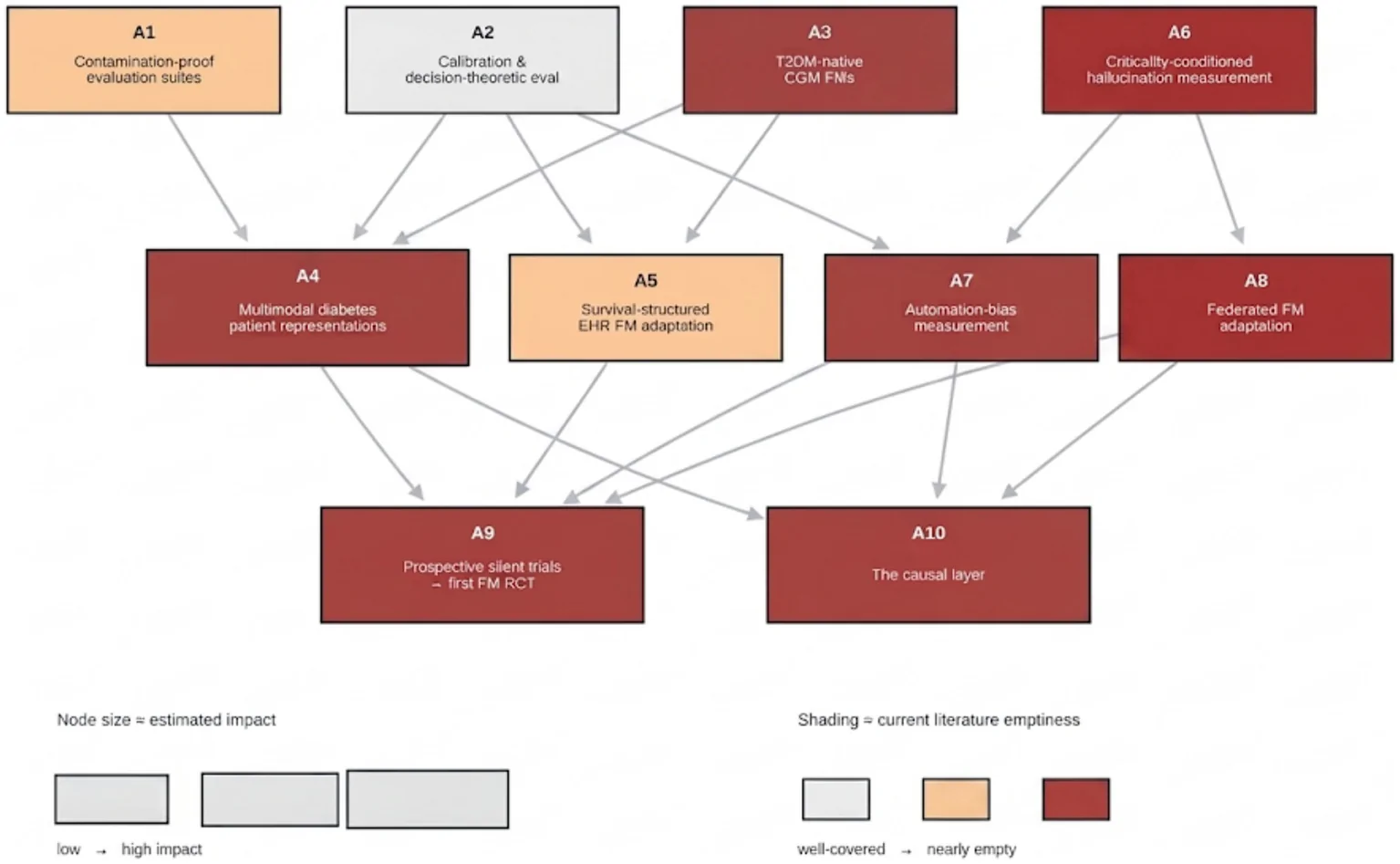
Research-agenda dependency graph (A1–A10). Arrows show prerequisite relationships among agenda items; node size is proportional to estimated impact and shading to current literature emptiness. Source: Authors’ qualitative estimates (Section 8).

## 9 Limitations

This review has eight limitations. (1) Coverage cutoff and time-sensitive negative findings. The literature window closes in December 2025; given the field’s monthly churn, contemporaneous work may already have filled specific gaps, most plausibly a registered prospective FM trial or a T2DM CGM pretraining study (items A3, A9). Every statement that a type of evidence is absent is bounded by the literature we reviewed and this cutoff (Section 2.1) and may already be out of date. (2) Narrative-review selection bias and databases. The literature was identified through purposive searching using explicit query strings (Section 2.2), with corpus construction involving dual-reviewer screening and consensus resolution; the principal studies are summarized descriptively in Table 3, but no record counts or agreement statistics are reported, and the search was not exhaustive. Scopus, Web of Science, DBLP, the ACM Digital Library, and Google Scholar were not searched, so relevant work indexed only there, or missed by purposive searching, may be absent. The evidence-level appraisal (Section 6.2) could shift with exhaustive systematic enumeration. However, the L1 vacancy is a structural property of the field, not a sampling artifact, since a single counterexample would falsify it. (3) Closed-model opacity. Parameter counts, corpus composition, and contamination status of closed-weight models are undisclosed; our analysis of them necessarily relies on published evaluations and is weaker in kind than our analysis of open models. (4) Metric heterogeneity. Cross-study metric comparisons (Tables 4–6) are qualitative by construction; we have deliberately avoided meta-analytic aggregation. (5) Authorship and clinical perspective. The authors are computer scientists with no clinical co-author, so the D1–D4 risk framing, the severity judgments in Table 2 and Figure 2, and the claims about clinical workflow and agenda feasibility (Sections 5.3, 7, and 8) are hypotheses for clinical review, not validated findings (Section 2.1). These elements are therefore offered as a proposal for clinical review and should not guide clinical practice until they are validated. Structured review by endocrinologists, ophthalmologists, and diabetes educators (for example, a Delphi or expert-panel process applied to the D1–D4 classes and Table 2), or clinical co-authorship, is the needed next step and a priority for follow-up work. (6) Grading and risk of bias. Evidence levels were assigned by the authors as an interpretive device, without formal reliability testing, and describe the validation ladder, not study quality, because formal risk-of-bias tools were not applied; the reasons for not applying PROBAST, QUADAS-2, and APPRAISE-AI are given tool by tool in Section 2.3. Some grading errors may remain, and the patterns in Section 6.2 should be read as approximate. (7) Language. Restricting the reviewed literature to English-language reports may omit relevant work, particularly regional diabetes-screening deployments. (8) Explainability. Explanation quality and faithfulness were not part of the analytic frame (Section 2.4); Section 4.5 is a conceptual comparison of interpretability across the three eras, not a systematic appraisal of explanation methods in the diabetes literature.

## 10 Discussion

The three-era taxonomy of Section 3 has a practical implication that the modality-by-modality survey of Sections 4–5 makes concrete: era-III capability gains and era-III evaluation readiness are decoupled, to different degrees in different modalities. Ophthalmic FMs sit closest to clinical translation because they inherit era-II’s regulatory precedent (IDx-DR) and a comparatively mature benchmarking culture; CGM and EHR FMs sit furthest because we found no comparable precedent or benchmarking culture for them; and clinical LLMs sit in between, capable of static question-answering but unevaluated on the dynamic, patient-specific decisions in which they are already being deployed. FMs in diabetes care are therefore not a single readiness question: funding, regulatory attention, and evaluation infrastructure should be allocated modality by modality, weighted toward the domains identified in Section 6.2 as furthest behind.

The safety analysis of Section 7 and the evaluation crisis of Section 6 are the same problem seen from two angles: a field without prospective, calibrated, subgroup-disaggregated evaluation cannot distinguish a safe FM from one that merely looks safe on benchmark-shaped tasks. Diabetes care makes the distinction unusually consequential because its deployment surface reaches directly into patient-facing dosing decisions (Section 1.2). The research agenda of Section 8 follows from this diagnosis: the priority items are not new architectures but the evaluation, grounding, and governance infrastructure that would let existing architectures be trusted. Whether the field closes this gap before or after wider clinical deployment is, in our view, the central open question for FMs in diabetes care over the coming several years.

## 11 Conclusion

The foundation-model transition reaches diabetes care as it reaches most of medicine: as a capability story riding on generic architectures, domain specificity concentrated in corpora, and evaluation inherited unchanged from a supervised-learning era, it no longer fits. This review maps the transition modality by modality: retinal pretraining that turns fundus photographs into general microvascular representations, temporal pretraining that makes CGM streams transferable across patients, sequence pretraining that makes the EHR a language, and LLMs that know diabetes guidelines well while being unable to guarantee a single safe dose recommendation. The label-efficiency dividend is consistently reported, and where labels are scarce, it supports an economic transition. But the evidence-level appraisal is unforgiving: across the era-III diabetes literature we reviewed, prospective validation is effectively absent as of December 2025, calibration reporting is exceptional, fairness disaggregation is rare, and we found no published report in which an FM was shown to change a diabetes outcome. The field’s center of gravity must therefore shift from building representations to building accountable pipelines: contamination-proof benchmarks, decision-theoretic evaluation, federated and privacy-audited adaptation, criticality-conditioned safety measurement, and (ultimately) the first randomized trial of an FM in a diabetes workflow. Diabetes, with its unmatched data density, label-expensive endpoints, regulatory precedent, and patient-facing risk surface, is the right disease in which to attempt it. FMs have made diabetes AI more capable. Whether they make it more useful is no longer a modeling question. It is the evaluation, grounding, and governance problem this review has tried to make precise.
